# Multi-ancestry genome-wide association study of cannabis use disorder yields insight into disease biology and public health implications

**DOI:** 10.1038/s41588-023-01563-z

**Published:** 2023-11-20

**Authors:** Daniel F. Levey, Marco Galimberti, Joseph D. Deak, Frank R. Wendt, Arjun Bhattacharya, Dora Koller, Kelly M. Harrington, Rachel Quaden, Emma C. Johnson, Priya Gupta, Mahantesh Biradar, Max Lam, Megan Cooke, Veera M. Rajagopal, Stefany L. L. Empke, Hang Zhou, Yaira Z. Nunez, Henry R. Kranzler, Howard J. Edenberg, Arpana Agrawal, Jordan W. Smoller, Todd Lencz, David M. Hougaard, Anders D. Børglum, Ditte Demontis, Veterans Affairs Million Veteran Program, J. Michael Gaziano, Michael J. Gandal, Renato Polimanti, Murray B. Stein, Joel Gelernter

**Affiliations:** 1 Division of Human Genetics, Department of Psychiatry, Yale University School of Medicine, New Haven, CT USA; 2 Department of Psychiatry, Veterans Affairs Connecticut Healthcare Center, West Haven, CT USA; 3 Department of Anthropology, University of Toronto, Mississauga, Ontario Canada; 4 Biostatistics Division, Dalla Lana School of Public Health, University of Toronto, Toronto, Ontario Canada; 5 Department of Epidemiology, University of Texas MD Anderson Cancer Center, Houston, TX USA; 6 Department of Genetics, Microbiology and Statistics, Faculty of Biology, University of Barcelona, Catalonia, Spain; 7 VA Boston Healthcare System, Massachusetts Veterans Epidemiology Research and Information Center, Boston, MA USA; 8 Department of Psychiatry, Boston University Chobanian and Avedisian School of Medicine, Boston, MA USA; 9 Department of Psychiatry, Washington University School of Medicine, Saint Louis, MO USA; 10 NIHR Biomedical Research Centre, Moorfields Eye Hospital NHS Foundation Trust and UCL Institute of Ophthalmology, London, UK; 11 Research Division, Institute of Mental Health, Singapore, Singapore; 12 Stanley Center for Psychiatric Research, Broad Institute of MIT and Harvard, Cambridge, MA USA; 13 Department of Psychiatry and Molecular Medicine, Zucker School of Medicine at Hofstra/Northwell, Hempstead, NY USA; 14 Center for Addiction Medicine, Department of Psychiatry, Massachusetts General Hospital, Boston, MA USA; 15 Harvard Medical School, Boston, MA USA; 16 Department of Biomedicine, Aarhus University, Aarhus, Denmark; 17 The Lundbeck Foundation Initiative for Integrative Psychiatric Research, iPSYCH, Aarhus, Denmark; 18 Mental Illness Research, Education and Clinical Center, Crescenz VAMC and Center for Studies of Addiction, University of Pennsylvania Perelman School of Medicine, Philadelphia, PA USA; 19 Departments of Biochemistry and Molecular Biology and Medical and Molecular Genetics, Indiana University School of Medicine, Indianapolis, IN USA; 20 Psychiatric and Neurodevelopmental Genetics Unit, Center for Genomic Medicine, Massachusetts General Hospital, Boston, MA USA; 21 Center for Precision Psychiatry, Department of Psychiatry, Massachusetts General Hospital, Boston, MA USA; 22 Center for Neonatal Screening, Department for Congenital Disorders, Statens Serum Institut, Copenhagen, Denmark; 23 Center for Genomics and Personalized Medicine, Aarhus, Denmark; 24 The Novo Nordisk Foundation Center for Genomic Mechanisms of Disease, Broad Institute of MIT and Harvard, Cambridge, MA USA; 25 Million Veteran Program Coordinating Center, VA Boston Healthcare System, Boston, MA USA; 26 Department of Medicine, Division of Aging, Brigham and Women’s Hospital, Boston, MA USA; 27 Departments of Psychiatry and Genetics, Perelman School of Medicine, University of Pennsylvania, Philadelphia, PA USA; 28 The Lifespan Brain Institute, Penn Medicine and the Children’s Hospital of Philadelphia, Philadelphia, PA USA; 29 Psychiatry Service, VA San Diego Healthcare System, San Diego, CA USA; 30 Department of Psychiatry and Herbert Wertheim School of Public Health, University of California San Diego, La Jolla, CA USA

**Keywords:** Behavioural genetics, Genome-wide association studies

## Abstract

As recreational use of cannabis is being decriminalized in many places and medical use widely sanctioned, there are growing concerns about increases in cannabis use disorder (CanUD), which is associated with numerous medical comorbidities. Here we performed a genome-wide association study of CanUD in the Million Veteran Program (MVP), followed by meta-analysis in 1,054,365 individuals (*n*_cases_ = 64,314) from four broad ancestries designated by the reference panel used for assignment (European *n* = 886,025, African *n* = 123,208, admixed American *n* = 38,289 and East Asian *n* = 6,843). Population-specific methods were applied to calculate single nucleotide polymorphism-based heritability within each ancestry. Statistically significant single nucleotide polymorphism-based heritability for CanUD was observed in all but the smallest population (East Asian). We discovered genome-wide significant loci unique to each ancestry: 22 in European, 2 each in African and East Asian, and 1 in admixed American ancestries. A genetically informed causal relationship analysis indicated a possible effect of genetic liability for CanUD on lung cancer risk, suggesting potential unanticipated future medical and psychiatric public health consequences that require further study to disentangle from other known risk factors such as cigarette smoking.

## Abstract (web summary)

Multi-ancestry genome-wide association meta-analyses identify risk loci for cannabis use disorder. Genomic structural equation modeling and genetic correlation analyses show overlap with several other traits, including impulsivity and psychopathology.

## Main

Cannabis is a psychoactive substance with a long history of use and dependence. Recently within the United States, 37 states have approved what is termed medical cannabis use, and 19 states, 2 territories and the District of Columbia allow possession of cannabis for recreational purposes. In Europe, only Malta has fully legalized recreational cannabis, although many other countries have decriminalized possession of small amounts of cannabis and have enabled medical allowances. It was recently legalized in Thailand but remains prohibited in many parts of Asia, the Middle East and South America. The status in many of these places may be subject to change in the near future. More than a third of individuals who use cannabis develop cannabis use disorders (CanUD), and evidence regarding the impact of legalization on escalating use and use disorders is mixed^1,2^. Substantial negative health outcomes associated with chronic cannabis use include various cancers associated with inhaling combustion products^3^, declines in cognitive capacity and motivation and increased schizophrenia (SCZ) risk^4,5^. Individual and societal complications that result from CanUD include decreased productivity and accidents related to intoxication^6^. The full range of risks and negative outcomes associated with cannabis use and CanUD may not be appreciated widely. Considering the gradually increasing permissiveness surrounding its use, understanding various sources of risk that influence CanUD is both necessary and timely.

In this Article, we combined genome-wide genotype data from the Million Veteran Program (MVP) with expanded samples from iPSYCH2^7,8^ and Mass General Brigham (MGB) BioBank^9^ and meta-analyzed these with the Psychiatric Genomics Consortium (PGC)/deCODE/iPSYCH1 study^7,10^. MVP, one of the largest biobanks in the world^11^, has enabled a substantial increase in power for genomic discovery by doubling the number of cases of European (EUR) ancestry available. By increasing sample numbers, we substantially increased the number of discovered loci and confirmed previous findings^7,10^. We also leveraged the ancestral diversity of the MVP to expand analyses of African ancestry individuals (AFR) and conducted genome-wide association studies (GWAS) analyses in Admixed American (AMR) and East Asian (EAS) ancestries. Linkage disequilibrium (LD) score regression (LDSC) can quantify variance explained by genetics and identify overlap between traits. This method is sufficient for EUR ancestries but not appropriate for some non-European and admixed ancestries. To solve this problem, we used cohort-derived covariate LDSC^12^ to calculate single nucleotide polymorphism (SNP)-based heritability in these populations, finding similar results among all ancestries. We conducted a transcriptome-wide association study (TWAS), which leverages annotations based on variant associations to changes in gene expression, in adult and fetal brain tissue to identify significant expression quantitative trait loci (eQTLs), using stratified LDSC to show enriched SNP-based heritability in fetal but not adult cortex. We also conducted Mendelian randomization (MR) analyses—an approach that uses genetic variations identified by GWAS as instruments to obtain an unbiased estimate of the effect of a trait of interest (here, CanUD) on outcomes—to examine causal relationships with chronic pain, lung cancer, physical activity and SCZ. Finally, we performed genomic structural equation modeling (gSEM)—a multivariate method for analyzing GWAS summary statistics to examine joint genetic architecture of traits—to understand the genomic relationships between cannabis use traits and other psychiatric and substance use disorder (SUD) traits. This work builds upon a decade of progress in the field^7,10,13–18^.

## Results

### GWAS

**Fig. 1:**
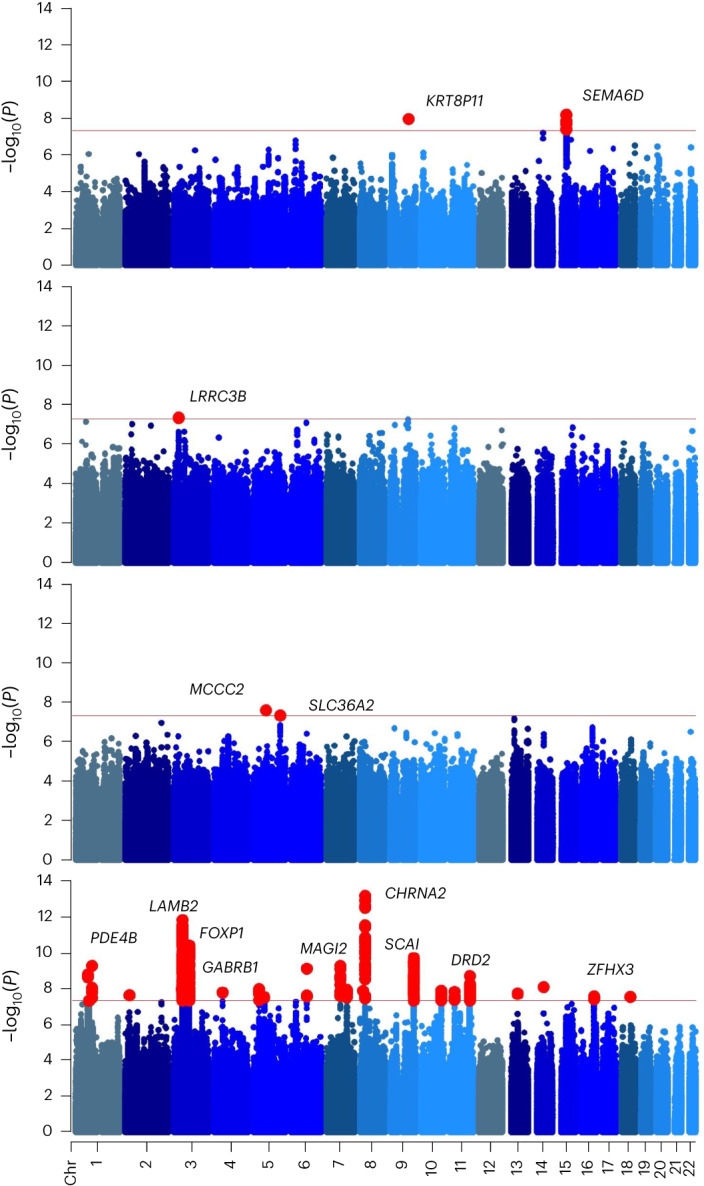
Stacked Manhattan plots depicting CanUD GWAS results from four ancestries tested. From top to bottom, 2 loci were identified in EAS ancestry, 1 for AMR, 2 for AFR, and 22 for EUR (red dots). Nearby genes are shown for orientation. *P* values were calculated with bidirectional Wald’s test. The field standard GWS threshold of *P* < 5 × 10^−^^8^ (horizontal red line) was used to determine significant associations. Other colors indicate different chromosomes. Chr, chromosome.

We assembled a total sample of 886,025 EUR participants across five datasets (Table 1; 42,281 cases and 843,744 controls) for GWAS meta-analysis of CanUD and identified 22 independent genome-wide significant (GWS) loci in this population. In the AFR meta-analysis of 123,208 participants across three cohorts (19,065 cases and 104,143 controls), we identified two GWS loci. In a cohort of 38,289 participants assigned using the broad AMR ancestry references (which include individuals recruited from several Latin American populations) in the MVP cohort (2,774 cases and 35,515 controls) we found one GWS locus, and in EAS ancestry references we identified two GWS loci. The lead signal for EUR was near *CHRNA2* (rs56372821, *P* = 7.3 × 10^−14^), which encodes cholinergic receptor nicotinic alpha 2 subunit, consistent with prior GWAS^7,10^; the lead SNP was identical to one prior study^7^. Findings for AFR include a SNP in an intron of *SLC36A2* (rs573117193, *P* = 4.9 × 10^−^^8^), which encodes a pH-dependent proton-coupled amino acid transporter for glycine, alanine and proline. The lead SNP in AMR was rs9815757 (*P* = 4.4 × 10^−^^8^). The lead SNP in EAS (rs78561048, *P* = 6.7 × 10^−^^9^) is intronic to *SEMA6D*, which encodes semaphorin 6D (Fig. 1 and Table 2). Several variants showed concordant direction of effect across all four stratified ancestral groups. Five additional loci were discovered in the multi-ancestry analysis: rs7003100 (intergenic), rs7029483 (130 kb upstream of *MTND2P8*), rs2627197 (intronic to *ENO4*), rs34438449 (40 kb downstream of *MIR5007*) and rs147144681 (intronic to *CHRNA3*).

**Table 1:** Demographics

Population	Cohort	Status	*n*	Totals	Effective
EUR	PGC+deCODE	Case	14,522	313,463	55,397
		Control	298,941		
	MVP	Case	22,260	445,847	84,594
		Control	423,587		
	iPSYCH2	Case	4,733	100,390	18,039
		Control	95,657		
	MGB	Case	456	24,544	1,790
		Control	24,088		
	Yale–Penn 3	Case	310	1,781	1,024
		Control	1,471		
	Total	Case	42,281	886,025	161,053
		Control	843,744		
AFR	PGC	Case	3,848	9,745	9,314
		Control	5,897		
	MVP	Case	14,946	112,526	51,843
		Control	97,580		
	Yale–Penn 3	Case	271	937	770
		Control	666		
	Total	Case	19,065	123,208	64,460
		Control	104,143		
AMR	MVP	Case	2,774	38,289	10,292
		Control	35,515		
EAS	MVP	Case	194	6,843	754
		Control	6,649		

PGC is not a single cohort but comprises several individual cohorts, as described^10^

**Table 2:** Lead SNP for each ancestral group

RSID	POS	Allele+	Allele−	EUR *P*	AFR *P*	AMR *P*	EAS *P*	Effect
rs7519259	1:66434743	A	G	**1.83** **×** **10**^**−**^^**9**^	*0.08*	*0.94*	0.31	+++−
rs6690119	1:73580964	T	C	**5.00** **×** **10**^**−**^^**8**^	*0.23*	0.57	*0.41*	++−+
rs1526480	1:91209986	T	C	**5.91** **×** **10**^**−**^^**10**^	*0.17*	0.61	*0.22*	−+−
rs719504	2:22918025	A	G	**2.53** **×** **10**^**−**^^**8**^	0.32	n/a	n/a	+−XX
rs184064410	3:43992164	T	C	**1.20** **×** **10**^**−**^^**8**^	n/a	*0.36*	n/a	+X+X
rs3774800	3:49334768	A	G	**1.72** **×** **10**^**−**^^**12**^	0.96	0.07	0.16	−+
rs17007864	3:70876858	T	C	**1.05** **×** **10**^**−**^^**9**^	*0.72*	0.82	*0.99*	++−+
rs726610	3:85551403	T	C	**4.29** **×** **10**^**−**^^**11**^	*0.13*	*0.23*	0.38	−+
rs201175241	4:47126053	G	GA	**1.77** **×** **10**^**−**^^**8**^	0.47	*0.05*	0.63	+−+−
rs56070621	5:30825684	A	T	**1.15** **×** **10**^**−**^^**8**^	*0.17*	0.99	*0.09*	++−+
rs159365	5:60500273	A	G	**3.33** **×** **10**^**−**^^**8**^	0.96	*0.5*	*0.84*	+−++
rs9344740	6:88619412	T	G	**8.34** **×** **10**^**−**^^**10**^	*0.01*	*0.4*	*0.26*	−−−−
rs62461183	7:77716309	T	C	**5.86** **×** **10**^**−**^^**10**^	*0.37*	*0.43*	0.85	+++−
rs2189010	7:114000000	A	G	**1.28** **×** **10**^**−**^^**8**^	n/a	*0.08*	n/a	+X+X
rs545943750	8:16059558	A	AT	**1.45** **×** **10**^**−**^^**8**^	n/a	n/a	n/a	−XXX
rs56372821	8:27436500	A	G	**7.27** **×** **10**^**−**^^**14**^	0.17	*0.93*	n/a	−+−X
rs10986600	9:128000000	T	C	**2.17** **×** **10**^**−**^^**10**^	*0.04*	*0.03*	0.25	+++−
rs200595759	10:119000000	T	TATA	**1.42** **×** **10**^**−**^^**8**^	n/a	n/a	n/a	−XXX
rs6484345	11:27996573	A	G	**1.63** **×** **10**^**−**^^**8**^	n/a	n/a	n/a	+XXX
rs34554234	11:113000000	G	GC	**2.20** **×** **10**^**−**^^**9**^	*0.18*	*0.41*	*0.74*	−−−−
rs80030908	13:55159898	A	G	**2.13** **×** **10**^**−**^^**8**^	*0.27*	*0.43*	n/a	+++X
rs62051488	16:72652784	A	C	**2.98** **×** **10**^**−8**^	*0.43*	*0.58*	*0.27*	−−−−
rs78561048	15:47805135	A	G	*0.38*	0.81	0.85	**6.71** **×** **10**^**−**^^**9**^	+–+
rs9815757	3:26809488	T	C	n/a	0.61	**4.36** **×** **10**^**−**^^**8**^	n/a	X+−X
rs574008891	5:70933608	T	C	n/a	**2.68** **×** **10**^**−**^^**8**^	n/a	n/a	X+XX
rs573117193	5:150713922	A	G	n/a	**4.90** **×** **10**^**−**^^**8**^	n/a	n/a	X+XX

*P* value listed from left to right. Effect indicates the effect allele for each ancestry. GWS results are bold; cross-ancestry concordant effect is marked by italicized *P* values. If SNP is not present, n/a is reported.

### LDSC

**Fig. 2:**
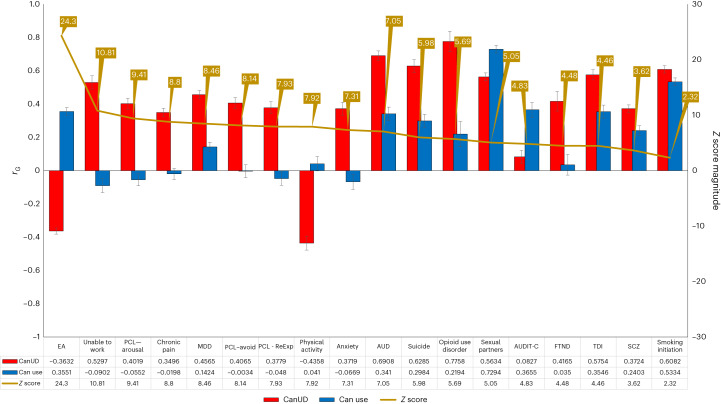
Genetic correlations. Comparison of genetic correlations between CanUD and cannabis use^18^. Left axis depicts the range of *r*_G_ between 1 and −1. Red bars and blue bars depict the *r*_G_ point estimate per trait for CanUD and cannabis use, respectively. Black error bars represent the standard error. Right axis displays the absolute magnitude of the *Z* statistic for the difference in *r*_G_ between CanUD and cannabis use (gold line). SNP-based heritability for each comparison trait is included in the table. EA, educational attainment; ReExp, re-experience; AUDIT-C, Alcohol Use Disorders Identification Test-Consumption; TDI, Townsend deprivation index.

Intergroup comparisons between EUR CanUD cohorts (MVP, PGC/deCODE, iPSYCH2) included in the meta-analysis yielded high genetic correlation, with *r*_G_ ranging between 0.71 and 0.87. Comparative analysis of CanUD and cannabis use traits with a range of psychiatric and nonpsychiatric traits revealed striking differences, with CanUD showing far stronger overlap with pathological and negative traits (Fig. 2). The largest magnitude difference was in educational attainment, which showed a positive correlation with cannabis use but a negative correlation with CanUD. Covariate LDSC was used to calculate SNP-based heritability within each ancestral group. Significant SNP-based heritability was identified for the three larger ancestries: EUR *h*^2^ = 6.7% (standard error (s.e.) = 0.017), AFR *h*^2^ = 8.1% (s.e. = 0.013), and AMR *h*^2^ = 18.0% (s.e. = 0.042). There was high variance and a high point estimate in AMR. LDSC was used to calculate genetic correlation between cannabis use dependence cohorts included in this meta-analysis and also within MVP phenotype definitions (Supplementary Table 1). Genetic correlations were calculated for 1,335 traits (Fig. 2 and Supplementary Fig. 2). The strongest observed positive correlations were related to smoking initiation and alcohol dependence, while the strongest negative correlations were with ages of first intercourse and smoking cessation.

### Cross-ancestry genetic correlation

Genetic correlations were calculated against available traits using POPCORN^19^ for CanUD in African ancestry and a selection of traits represented in Fig. 2. When compared to the same traits in EUR, there is no significant difference across ancestries (Supplementary Fig. 3).

### Mendelian randomization

Multi-site chronic pain had a unidirectional causal effect on CanUD (inverse variance-weighted (IVW) *β* = 0.46, *P* = 2.90 × 10^−5^). There was a bidirectional causal effect of CanUD and SCZ (SCZ→CanUD IVW *β* = 0.17, *P* = 2.07 × 10^−5^, CanUD→SCZ IVW *β* = 0.17, *P* = 0.01). CanUD showed a unidirectional effect on lung cancer (IVW *β* = 0.18, *P* = 0.006) (Supplementary Fig. 1 and Supplementary Tables 5–13).

### Conditional analysis

For EUR, we performed a multi-trait conditional and joint analysis (mtCOJO) of CanUD conditioned on two smoking traits from the GWAS and Sequencing Consortium of Alcohol and Nicotine use study to investigate potential confounding effects^20^. Two different datasets were used: smoking initiation and cigarettes per day. Individual runs were performed for the two cigarette smoking traits. A proportion of 18 of 22 original lead SNPs remained in the dataset following conditioning on smoking initiation (meaning they matched with variants in the conditioning data). For two out of four remaining SNPs, there were proxy SNPs in LD with each lead SNP showing GWS *P* values. Only rs545943750 and rs184064410 were excluded after conditioning due to missingness in the smoking data, leaving 20 of 22 lead loci from the CanUD GWAS available in the conditional analysis. All 20 remained GWS following conditioning. The results were similar with CanUD conditioning on cigarettes per day, with the same 20 lead loci remaining GWS after conditioning. Conditional analysis with smoking initiation or cigarettes per day did not substantially alter the magnitude of the lead *CHRNA2* association (*P*_cond_ = 2.14 × 10^-14^). We used these summary statistics conditioned on cigarette smoking initiation to re-test the causal relationship between CanUD and lung cancer, and while the signal attenuated, it was still significant (IVW *β* = 0.2, *P* = 0.0025). The conditional analysis with cigarettes per day, however, removed the effect of CanUD on lung cancer (*P* = 0.79).

### Multi-trait analysis of GWAS

Considering the high genetic correlation of CanUD with alcohol use disorder (AUD) and the Fagerström Test for Nicotine Dependence (FTND), we conducted an multi-trait analysis of GWAS (MTAG) analysis that identified 34 lead SNPs at 26 genomic risk loci, including four novel loci compared to the EUR meta-analysis, at *P* < 5 × 10^−8^ for CanUD (Supplementary Fig. 5 and Supplementary Table 14) when combined with AUD and FTND. The GWAS-equivalent sample size for CanUD was 200,762, augmenting the meta-analysis effective sample size of 161,053 by 20%. Ten genomic risk loci were significant (or in LD with significant variants) in both the GWAS and MTAG analyses. The remaining 16 significant variants were LD independent. The effect size of eight of the 26 significant SNPs in the MTAG analysis was significantly smaller than those obtained from the original GWAS (Supplementary Table 15), suggesting specificity to CanUD.

### Transcriptome-wide association study

**Fig. 3:**
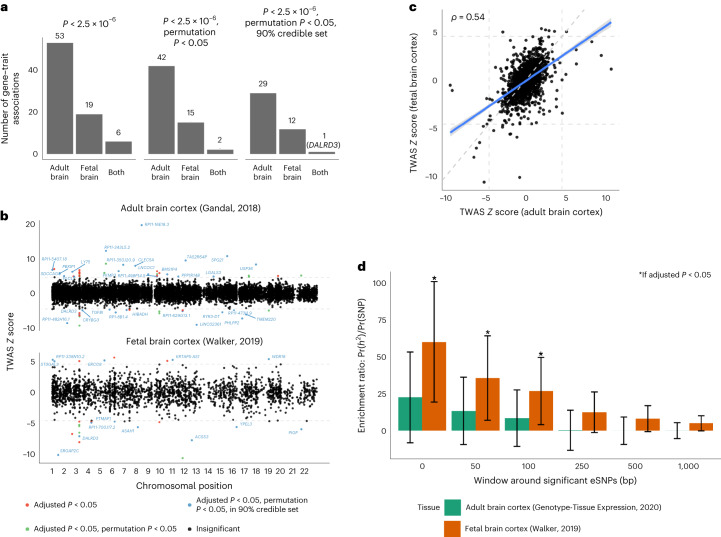
TWAS and tissue enrichment of the EUR CanUD GWAS variants. **a**, Number of TWAS gene–trait associations for adult and fetal brain frontal cortex, across significance thresholds. **b**, Miami plot of TWAS *Z* scores across analyses with adult (top) and fetal (bottom) brain frontal cortex. Genes are colored red if the gene passes transcriptome-wide significance, green if the gene additionally passes permutation testing and blue if the gene is estimated to be in the 90% credible set at the locus. All genes in blue are labeled. The horizontal line indicates threshold statistical significance following multiple testing correction. **c**, Scatterplot of standardized effect sizes (TWAS *Z* scores) across adult and fetal brain frontal cortex. A total of 10,722 genes were tested in adult tissue, while 2,293 genes were tested in fetal tissue. The dashed line indicates the expected relationship if no enrichment for adult or fetal brain. **d**, Enrichment ratio point estimate of proportion of SNP-based heritability explained by annotation and proportion of SNPs in annotation. Error bar shows a 95% Wald-type confidence interval. Bars marked with an asterisk indicate an enrichment ratio >1 at Benjamini–Hochberg adjusted *P* < 0.05.

In TWAS analyses, 59 and 25 genes were detected (*P* < 2.5 × 10^−^^6^) using adult and fetal brain frontal cortex expression, respectively, with six genes in common (Fig. 3a). We tested these genes by permutation test, leaving 44 and 17 genes using adult and fetal models, with two genes in common (Fig. 3a). For the remaining genes within 1 Mb of one another, we applied gene-level probabilistic fine-mapping. In the end, we detected 36 and 15 genes using the adult and fetal models, which form 90% credible sets (with 90% estimated probability of containing the causal variant) that explain the corresponding genetic associations (Fig. 3a, b). These sets contained only one gene in common: DALR Anticodon Binding Domain Containing 3 (*DALRD3*) (Fig. 3a, b). The observed gene associations included four distinct GWAS loci: 3p21.31 (gene detected in adult and fetal brain cortex: *DALRD3*), 5q12.1 (fetal*: ERCC8*), 11q23.2 (adult: *RP11-629G13.1*) and 16q22.2 (adult: *PHLPP2*). Protein functions of these genes are described in the Discussion below. The remaining set of genes identifies 38 candidate novel genetic loci associated with CanUD, with potential underlying transcriptomic mechanisms in either adult or fetal brain cortex (Supplementary Table 3).

### Partitioned SNP-based heritability

Standardized TWAS effect sizes estimated using adult and fetal brain frontal cortex expression models showed moderate correlation (Spearman’s *ρ* = 0.54, *P* < 2.2 × 10^−^^16^; Fig. 3c). Accordingly, we next estimated the SNP-based heritability enrichment in adult and fetal brain cortex eQTL. Using LDSC, we estimated enrichment ratios for SNP-based heritability using different windows around expression SNPs for expression genes. We detected significant enrichments only for fetal brain frontal cortex expression SNPs at windows of 0 bp, 50 bp and 100 bp. In general, fetal brain frontal cortex eQTLs were far more enriched for CanUD trait heritability than adult brain cortex eQTLs (Fig. 3d).

### gSEM

Using exploratory factor analysis (EFA), a four-factor model fit the data best, with the cumulative variance explained being 0.789, distributed relatively evenly across the four factors, with each accounting for between 22.7% and 29.2% of the overall variance explained (factor 1 of 0.23, factor 2 of 0.19, factor 3 of 0.18 and factor 4 of 0.18). Each of the four factors had high sums of square (SS) loadings (factor 1 SS of 3.5, factor 2 SS of 2.9, factor 3 SS of 2.8 and factor 4 SS of 2.7).

**Fig. 4:**
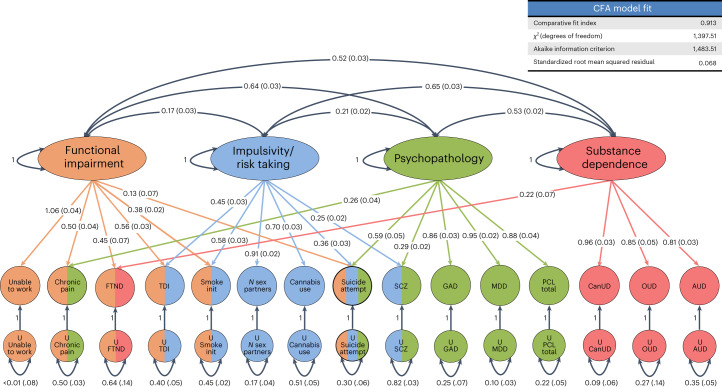
gSEM. gSEM was used to cluster 14 traits correlated with CanUD. Exploratory and confirmatory factor analysis indicated four factors fit the data best. Factors fit mostly into categories that we designated as functional impairment (factor 1), impulsivity and risk taking (factor 2), psychopathology (factor 3) and substance dependence (factor 4). CanUD fit best (and strongly) in the substance dependence cluster. FTND fit into factors for functional impairment and substance dependence. Suicide attempts fit into functional impairment, impulsivity and risk taking, and psychopathology. Numbers outside the parenthesis are correlation coefficients between factors. Numbers inside parenthesis are stadard errors of means. Smoke init, initiation of regular smoking; GAD, generalized anxiety disorder; OUD, opioid use disorder.

Using confirmatory factor analysis (CFA) to evaluate the four-factor model that allowed all factors to intercorrelate had a comparative fit index of 0.913, a standardized root mean square residual of 0.068, a chi-squared value of 1397.5 and an Akaike information criterion of 1483.5. Traits loading most strongly on factor 1 included ‘Unable to work’ (loading of 1.06 ± 0.04), Townsend deprivation index (loading of 0.56 ± 0.03), chronic pain (loading of 0.50 ± 0.04) and FTND (loading of 0.45 ± 0.07). Traits loading most strongly on factor 2 included number of sex partners (loading of 0.91 ± 0.02), cannabis use (loading of 0.70 ± 0.03) and initiation of regular smoking (loading of 0.58 ± 0.03). Psychiatric traits loaded most strongly on factor 3 and included major depressive disorder (MDD) (loading of 0.95 ± 0.02), post-traumatic stress disorder (PTSD) checklist score (PCL) total (loading of 0.88 ± 0.04), generalized anxiety disorder symptoms (loading of 0.86 ± 0.03), suicide attempt (loading of 0.59 ± 0.05) and SCZ (loading of 0.29 ± 0.02). SUD traits loaded most strongly on factor 4 and included CanUD (loading of 0.96 ± 0.03), opioid use disorder (loading of 0.85 ± 0.05) and AUD (loading of 0.81 ± 0.03). There were moderate correlations between factors 2 and 4 (*r* = 0.65), factors 1 and 3 (*r* = 0.64), factors 1 and 4 (*r* = 0.52) and factors 3 and 4 (*r* = 0.53). All correlations and loadings are summarized in Fig. 4.

## Discussion

Recently, cannabis use has been legalized in various US states and elsewhere without fully examining the health consequences of individual or societal risks. An epidemiologic survey conducted by the National Survey on Drug Use and Health in the United States identified a past-year cannabis use prevalence of 17.5%, an increase from 11.0% in 2002, and 1.8% with CanUD, the same percentage recorded in 2002. Usage varies worldwide, with many regions of high prevalence^21^.

The findings we report here add to our understanding of CanUD biology on many levels. First, we greatly increased the available sample size for genomic analysis, mostly by incorporating MVP data, and identified multiple novel risk loci in four populations, improving on previous results in EUR by more than an order of magnitude and presenting the first genetic discoveries in the other populations studied. Using the GWAS data, we then showed overlapping genetic liability to other traits. Next, investigating how genetic variation underlying CanUD influences fetal brain gene expression, the brain in particular showed significant enrichment for SNP-based heritability. Essentially, SNPs that influence fetal brain gene expression explain a greater proportion of CanUD phenotypic variance than the overall GWAS association of all SNPs. We investigated the overlapping and shared underlying genetic architectures of several different traits and employed MR to demonstrate putative causal relationships between outcomes with substantial impact on human health, including an association with lung cancer risk. Cannabis is frequently consumed using methods involving inhaling combustion products, potentially exposing users to risks similar to those found in smoking other substances such as tobacco. Indeed, some of the shared genetic risk between CanUD and tobacco smoking may relate to propensity to smoke per se, independent of substance, a hypothesis that we currently lack the power to evaluate.

We identified 22 significant loci, most of them novel, for CanUD in EUR. We also replicated findings in *CHRNA2* (meta *P* = 7.3 × 10^−^^14^, MVP only *P* = 1.1 × 10^−^^5^) and *FOXP2* (meta *P* = 1.7 × 10^−^^8^, MVP only *P* = 2.0 × 10^−^^3^), with triple the effective sample size of the largest of those studies^10^, demonstrating once again the stability of GWAS findings as sufficient sample size and power to discover new loci are reached^22,23^. We discovered GWS loci in four ancestral groups: EUR, AFR, AMR and EAS. In AFR, two independent SNPs were associated on chromosome 5. The first (rs574008891) was within an intron of the gene that encodes methylcrotonyl-CoA carboxylase subunit 2 (*MCCC2*). The other significant locus (rs573117193) mapped to an intron in the solute carrier family 36 member 2 (*SLC36A2*) gene. These specific variants are absent in the other ancestries studied. For AMR, the one risk locus was rare (rs9815757, minor allele frequency (MAF) 0.1%) and mapped in an intergenic region downstream of leucine rich repeat containing 3B (*LRRC3B*). Finally, for EAS, one locus was associated with CanUD: rs78561048, near semaphorin 6D (*SEMA6D*). Follow-up analysis in larger samples is needed to assess the robustness of findings, particularly in AMR and EAS. Several variants showed concordant direction of effect across all four stratified ancestral groups (Table 1). For instance, rs10986600, significantly associated in EUR on chromosome 9, was nominally significant (*P* < 0.05) with same effect direction in AFR (0.04) and AMR (0.03) and significant in the multi-ancestry meta-analysis. This intronic variant of the protein phosphatase 6 catalytic subunit (*PPP6C*) is an eQTL for *PPP6C*, a gene linked to various cancers, including skin melanoma and lung squamous cell carcinoma. Multi-ancestry meta-analysis revealed an additional five loci not identified in the stratified analyses. Among them, the lead SNP on chromosome 15, rs147144681, which maps to an intron of the cholinergic receptor nicotinic alpha 3 subunit (*CHRNA3*) gene, is particularly noteworthy; as reported above, variation in *CHRNA2* was among the first variants associated with CanUD and was replicated here. This suggests potential convergence involving the cholinergic system broadly and nicotinic receptors, specifically in the underlying etiology of CanUD. While nicotinic receptors are also associated with tobacco smoking-related traits^24^, the relative pattern of association for those traits is different from the observations for CanUD—for many smoking-related traits, a chromosome 15 nicotinic receptor cluster is associated with orders of magnitude greater support than other variants, including other nicotinic receptors; for CanUD, *CHRNA2* is consistently the strongest association, also by orders of magnitude. We conducted conditional analysis for *CHRNA2* and found the conditional *P* value remained robust following conditioning on smoking initiation^20^ (*P*_cond_ = 4.6 × 10^−^^14^). This replicates similar analyses performed by Demontis et al.^7^ and Johnson et al.^10^, which showed conditioning on smoking did not affect the CanUD association at this variant. Several other loci near cholinergic receptor subunit genes previously identified for smoking are not significant in our analysis of CanUD (*CHRNA4*, rs13036436, smoking *P* = 1.1 × 10^−^^29^, CanUD *P* = 0.97; *CHRNA5*, rs667282, smoking *P* = 9.9 × 10^−^^25^, CanUD *P* = 0.043). Conversely, the *CHRNA3* variant we find associated with CanUD is not significant for smoking (rs147144681, smoking *P* = 0.0033, CanUD *P* = 3.3 × 10^−^^8^) (ref. ^20^).

Genetic correlations were calculated for 1,335 traits to identify genetic overlap with CanUD. Some traits with significant *r*_G_ were tested for causal inference based on a combination of significant genetic correlation and a prior interest in phenotype (physical activity, multi-site chronic pain, Alzheimer’s disease and SCZ). We identified a bidirectional causal relationship between CanUD and SCZ. At the same time, the MR Egger analysis indicated this was not due to horizontal pleiotropy. This supports similar findings reported previously, confirming previous genetic–epidemiologic studies^25^ and verifying an important public health risk associated with CanUD. To highlight differences between cannabis use and CanUD, we compared the pattern of genetic correlations across 18 traits, which showed striking differences. CanUD was much more closely associated with psychopathology, recapitulating a general pattern seen with other comparisons of SUD and use traits^26^. For example, while we observed a substantial negative correlation between CanUD and educational attainment, cannabis use was associated with greater educational attainment. POPCORN was used to generate a cross-covariance score to allow for comparison of traits across ancestries using genetic correlations for EUR and AFR groups (Supplementary Fig. 3). We found a striking similarity for cross trait comparisons for both groups, indicating a similar underlying genomic architecture. This finding supports the possibility that some findings uncovered so far for EUR individuals, recruited in vastly greater numbers for genetic study, will provide some degree of generalizability across human populations.

Chronic pain may be a factor driving CanUD in some individuals, with significant unidirectional evidence for a causal effect of chronic pain^27^ on CanUD in the MR analysis. Cannabis use has been proposed as a treatment for chronic pain, and there are several clinical trials in progress^28^. This MR observation suggests that there may be merit in cannabis as a treatment for at least some kinds of pain. The small overall beneficial effect observed requires so many individuals to be treated that harmful effects (such as increased CanUD) also become a significant factor^29^. Our MR results suggesting that chronic pain has a causal influence on CanUD emphasize the need for follow-up investigations that address whether greater consideration should be given to the adverse effects, rather than just the therapeutic effects among individuals receiving cannabis-based medicines. A similar question arises with opioids, which although often prescribed for pain, can also cause great harm^30^: namely, what level of risk of CanUD is acceptable given cannabis’ potential to improve quality of life and reduce opioid exposure in chronic pain patients? Our results suggest that harms such as dependence and consequences, reflected in underlying genetics of the trait, may need to be weighed against the potential benefits of cannabis treatment for chronic pain. Future studies should consider this novel relationship to pain^31^ and clinical efficacy trials are underway.

Cigarette smoking substantially increases the risk of many forms of cancer, including lung cancer, through numerous well-studied mechanisms with established literature dating back more than 60 years^32^. The influence of cannabis on cancer risk is less well understood; it should be anticipated that these combustion products could have harmful pulmonary impacts—indeed, it would be surprising if smoking tobacco, but not smoking cannabis, increased cancer risk. MR yielded evidence for a unidirectional causal effect of CanUD on lung cancer. This result was robust to conditioning on data from the largest available smoking initiation GWAS but not conditioning on cigarettes per day, both traits that also have causal relationships with lung cancer but far more robust genetic instruments to evaluate this relationship. We do not currently have a way to assess genetic variation associated with the route of cannabis administration, but combustion is by far the most common method in the MVP and other cohorts studied. Given the trend toward increased legalization and usage, this apparent causal association needs to be monitored as it may have profound and underappreciated public health consequences. As the causal relationship with CanUD was not robust to conditioning on cigarettes per day, one probable explanation may be that there is horizontal pleiotropy between these traits in their influence on lung cancer.

Four GWS loci overlapped with TWAS prioritization from the EUR meta-analysis, using eQTL integration from samples of adult^33^ and fetal^34^ cortical tissue. These were *DALRD3* (both fetal and adult), *ERCC8* (fetal), *RP11-629G13.1* (adult) and *PHLPP2* (adult). The *DALRD3* protein product, a DALR anticodon binding domain, forms a complex with the product of *METTL2B*. Nonsense mutations in *DALRD3* are associated with developmental delay and early-onset epileptic encephalopathy^35^. *ERCC8* encodes the excision repair 8, CSA ubiquitin complex subunit, which plays a role in DNA repair and is associated with the developmental disorder Cockayne syndrome^36^, as well as breast, esophageal and other cancers^37,38^. *RP11-629G13.1* is a long noncoding RNA associated with downregulation of *NCAM1* gene expression in multiple myeloma patients^39^. Significant partitioned SNP-based heritability was observed in fetal but not in the adult cortex, with 4.36% of trait SNP-based heritability explained by 0.12% of the total SNPs near fetal frontal cortex eQTLs. Only 1.77% of CanUD SNP-based heritability was explained using 0.13% of the total SNPs near adult cortex eQTLs. Fetal development may play a role in SUD susceptibility^40^, and substance use can influence fetal development during pregnancy and health outcomes during childhood^41^. Although exogenous exposure to cannabis may not occur until years or decades after birth, enriched fetal SNP-based heritability in this study argues a possible role for genetic effects on CanUD in the developing brain independent of exposure. SCZ risk is also modulated by risk factors during fetal development^42^ and genetic^43^ and environmental effects (including maternal food deprivation in the first trimester of pregnancy^44^). Temporal convergence of the initiation of genetic risk effects for both SCZ and CanUD, if validated experimentally, would provide insight into the genetic relationship between these disorders and could relate to a mechanism for the bidirectional risk relationship between cannabis use and SCZ.

gSEM was used to contextualize summary statistics from this project with those from other published GWAS studies. Exploratory and confirmatory factor analyses showed that four factors provide the best fit for the 14 correlated traits included in the analysis. Factors fit mostly into categories that relate to functional impairment (factor 1), impulsivity and risk taking (factor 2), psychopathology (factor 3) and substance dependence (factor 4). CanUD fit best (and strongly) in the substance dependence cluster (factor 4). FTND fit into factors for functional impairment and substance dependence. Suicide attempts fit into functional impairment, impulsivity/risk taking and psychopathology. This is consistent with research showing overlapping pathologies within addiction and shared genetic risk factors between them^45^.

This study has limitations. The use of electronic health records allows for a large sample of CanUD cases but limits the assessment of subdiagnostic cannabis use in controls. Although we accounted for subdiagnostic cannabis users by excluding them from controls when information was available, these are probably underreported. Future studies of individuals with ascertained cannabis use who do not meet criteria for CanUD would provide more insight into the specific genetic liability to dependence. As the traits of interest were gathered from previously published reports or queries of electronic health records (EHRs) for diagnostic codes, we did not have information regarding tetrahydrocannabinol (THC) blood levels or information on the potency of cannabis at each exposure. If these data were available, study of effects on cannabis potency on dependence and comorbidities would be of great interest. We identified a causal relationship between multi-site chronic pain and CanUD. As pain is a complex trait and different type of pain may interact differently with CanUD, our finding for multi-site chronic pain is not sufficient to draw conclusions about the interaction between CanUD and specific kinds of pain or pain syndromes. Our definition of CanUD was based on any report of abuse or dependence either as an inpatient or outpatient. Participants in this study span a period of changing legal status and increasing use of marijuana, a major secular trend. Given the age of the participants (Supplementary Table 16) and expected time from initial exposure to the development of a use disorder, nearly all participants would have been exposed to cannabis before legalization. The TWAS study did not include ascertainment for CanUD in the individuals who donated brain tissue used for analysis. We discovered GWS loci in ancestral groups, but AFR, AMR and EAS sample sizes were small compared to EUR. We did not perform MR or TWAS analyses in non-European samples because available GWAS and eQTL datasets are still limited in non-European ancestry populations, and cross-ancestry analyses carry risk of biases due to differences in the underlying LD structure between ancestries. More studies are needed of individuals of diverse ancestries to replicate these findings, estimate their robustness and ensure that the benefits provided by these studies are available to all people.

This is the largest genetic study of CanUD so far, including data from multiple international cohorts in more than one million participants and comprising four ancestral groups. We replicate two prior GWS findings while identifying 25 novel loci, and we leverage these novel data to investigate genetic overlap with other traits. We identify a clear difference between cannabis use and CanUD, with genetic liability to CanUD being much more closely associated with psychopathology and disability. We found greater heritability enrichment in fetal than adult brain tissue, supporting an important role of development in laying the biological basis for CanUD. We used MR to assess causal relationships and found evidence of bidirectional causal effects between CanUD and SCZ and unidirectional effects of multi-site chronic pain on CanUD, and of CanUD on lung cancer. Finally, using gSEM, we found that CanUD loads on a latent factor with other substance dependence traits, consistent with clinical observation, genetic epidemiology and prior genetic studies of other SUD traits. In particular, we highlight the possible relationship revealed herein between CanUD and lung cancer risk. This study yields new insights into the genetic architecture of CanUD and how this risk interacts with traits crucial to public health and raises important concerns regarding the potential adverse consequences of the secular trend toward increased cannabis use consequent to legalization.

## Methods

### Inclusion and ethics statement

We included researchers from the iPSYCH biobank and the PGC, who played a role in study design. This research was not restricted or prohibited in the setting of any of the included researchers. All studies were approved by local instituational research boards and ethics review committees. MVP was approved by the Veterans Affairs central instituational research board. We do not believe our results will result in stigmatization, incrimination, discrimination or personal risk to participants.

### Cohorts

We used data release version 4 of the MVP. Linked and de-identified EHRs were queried using the Veterans Affairs Informatics and Computing Infrastructure to identify individuals with International Classification of Disease (ICD) codes for cannabis dependence or cannabis abuse (together, CanUD) (Supplementary Tables 2 and 3). The range of diagnosis dates was between May 1992 and December 2019. Two classifications were investigated: (1) cases identified by at least two separate outpatient visits or any number of inpatient visits to a US Veterans Affairs (VA) medical center for CanUD and (2) cases identified by at least one inpatient or outpatient visit for CanUD. Genetic correlation analysis indicated that these traits were almost identical from a genetic perspective (*r*_G_ = 0.99) and SNP-based heritability (*h*^2^) was not statistically different (definition 1, *h*^2^ = 0.075, s.e. 0.0053, *z* = 14.1; definition 2, *h*^2^ = 0.087, s.e. 0.0062, *z* = 14.0; *P*_diff_ = 0.14), so case definition per the second classification was retained for further analysis (that is, at least one inpatient or outpatient visit). All individuals diagnosed under the first disease definition were also diagnosed under the second more inclusive definition. Controls were defined as individuals without any VA EHR ICD codes for cannabis dependence, cannabis abuse or cannabis use (cannabis use codes included in ICD-9: 305.29 and included in ICD-10: F12.90, F12.920, F12.921, F12.922, F12.929, F12.93, F12.950, F12.951, F12.959, F12.980, F12.988 and F12.99). The PGC cohort was as previously described and was made up of 16 cohorts with varying phenotype definitions and ascertainments^10^. A leave-one-out analysis was performed to remove the iPSYCH1 sample, leaving 18,370 cases and 304,838 controls for European and African ancestries in the remaining PGC/deCODE sumstats. An updated expanded iPSYCH2 cohort was then added via meta-analysis (4,733 cases and 95,657 controls, all EUR). We also included samples from MGB Biobank (456 cases and 24,088 controls, all EUR) and new data from the Yale–Penn cohort^46^ beyond the individuals already included in the PGC study (an additional 310 cases and 1,471 controls for EUR, and 271 cases and 666 controls for AFR). Table 1 gives numbers for each cohort.

### MVP genotyping, imputation, quality control, and GWAS and meta-analysis

Genotyping and imputation of MVP participants has been described previously^11^. Briefly, a customized Affymetrix Axiom Array was used for genotyping. MVP genotype data for biallelic SNPs were imputed using Minimac4 and a reference panel from the African Genome Resources panel by the Sanger Institute. Indels and complex variants were imputed independently using the 1000 Genomes (1KG) phase 3 panel and merged in an approach similar to that employed by the UK Biobank. Designation of broad ancestries was based on genetic assignment with comparison to 1KG reference panels^47^.

MVP GWAS was conducted using logistic regression in PLINK 2.0 using the first ten positive controls, sex and age as covariates. Variants were excluded if call missingness in the best-guess genotype exceeded 20%. Alleles with MAF <0.1% were excluded in EUR, AFR and AMR. Alleles with MAF <1% were removed from EAS due to smaller sample size. The MVP data represented the largest and most diverse cohort with 22,260 cases and 423,587 controls (EUR), 14,946 cases and 97,580 controls (AFR), 2,774 cases and 35,515 controls (AMR) and 194 cases and 6,649 controls (EAS) (Table 1). GWAS meta-analyses in the PGC datasets of the deCODE and PGC samples were conducted as previously described, although a leave-one-out analysis was conducted to remove data from iPSYCH1 so that a larger cohort could be independently analyzed^10^. This leave-one-out PGC meta-analysis contained 14,522 EUR cases and 298,941 controls and 3,848 AFR cases with 5,897 controls. This study includes new genotypes from iPSYCH (referred to as iPSYCH2), and all iPSYCH data (iPSYCH1 + 2) has been reprocessed. Pre-imputation quality control and imputation were performed on genotypes from the full set of genotyped individuals for iPSYCH1 and iPSYCH2 separately, using standard procedures for GWAS data. The iPSYCH1 samples were genotyped in 23 genotyping waves and thus additional steps were taken to eliminate potential batch effects. Only variants present in more than 20 waves and with no significant association with wave status were retained. Imputation was done using the pre-phasing/imputation stepwise approach implemented in EAGLE v2.3.5^48^ and Minimac^49^, using the Haplotype Reference Consortium^50^ panel v1.0. GWAS of 4,733 EUR cases and 95,657 controls and was done on a merged set of best-guess genotypes with MAF >0.01 and imputation info score >0.8 (in both iPSYCH1 and iPSYCH2) using logistic regression with appropriate covariates (age, sex, psychiatric diagnoses (attention deficit hyperactivity disorder, autism spectrum disorder, SCZ, bipolar disorder and MDD), first ten positive controls and iPSYCH cohort of origin). A new Yale–Penn tranche was analyzed using PLINK 1.9 in unrelated individuals not previously included in any other GWAS or meta-analysis. This contributed 310 cases and 1,471 controls (EUR) and 271 cases and 666 controls (AFR). Finally, MGH Partners BioBank^51^ contributed 456 cases and 24,088 controls (EUR).

EUR cohorts were combined in a GWAS meta-analysis (Table 1). For AFR, we performed meta-analysis between the MVP, PGC and Yale–Penn cohorts. For AMR and EAS, only MVP included data so no meta-analysis was possible within these ancestries. GWAS meta-analyses were conducted using inverse variance weighing in METAL^52^ for both EUR and AFR. For within-ancestry meta-analyses, there were 42,281 EUR cases with 843,744 controls, and 19,065 AFR cases with 104,143 controls. The multi-ancestry meta-analysis^53^ included 1,044,620 total participants of EUR, AFR, AMR and EAS ancestries. Sex-stratified analysis was conducted in the only cohort available individual GWAS for the analysis—the MVP (Supplementary Fig. 7).

### LDSC and SNP-based heritability

LDSC was used to calculate SNP-based heritability on the liability scale, using a lifetime population prevalence^54^ of 2% and a sample prevalence of 5% for EUR, 13.2% for AFR, and 7.2% for AMR within the MVP^55^. We used the lifetime population prevalence reported in the PGC/deCODE/iPSYCH1 cannabis paper^10^ for comparability. Typically, calculating SNP-based heritability depends on reliable reference ancestry to account for nonindependence of some variance due to LD. This is easily done for EUR, but admixed non-European ancestries pose a statistical challenge. Covariate LDSC^12^ uses sample covariates such as those derived from principal components analysis (a dimension reduction technique that produces eigenvalues for each variant) carried out in the study sample to adjust LD scores to enable calculation of SNP heritability in each ancestry using sample-specific LD scores. LDSC as implemented by the Complex Traits Genomics Virtual Lab^56^ was used to estimate genetic correlations^57^ to identify common genetic architecture across all 1,335 traits available for comparison. Additionally, LDSC was used to compare genetic correlations between CanUD and cannabis use (from a previously published study^18^).

### Cross-ancestry genetic correlation

POPCORN^19^ was used to generate cross-ancestry covariance scores using 1KG reference panels from EUR and AFR. This method was applied to calculate genetic correlations between the AFR CanUD generated in this study against traits from Fig. 2 that had available allele frequencies and *n* count.

### Mendelian randomization

Several traits with significant genetic correlation with CanUD and high public health importance were selected for follow-up MR analysis in EUR ancestry datasets (‘type of physical activity in the last four weeks = none’, multi-site chronic pain, Alzheimer’s disease, SCZ and lung cancer). These traits were first tested for polygenic overlap with CanUD; one trait did not survive this step (Alzheimer’s disease), and the remaining three traits moved on to MR analysis. MR was conducted using the TwoSampleMR package in R Studio^58^. We conducted MR Egger analysis to test for the effect of horizontal pleiotropy.

### Conditional analysis

mtCOJO was carried out to study possible confounding of smoking for CanUD. GWAS summary statistics for smoking initiation and cigarettes per day from the GWAS and Sequencing Consortium of Alcohol and Nicotine use Phase 2 study of EUR ancestry were used for smoking^20^. The CanUD (target trait) GWAS data were conditioned on smoking initiation and cigarettes per day (covariate traits) GWAS data individually using the Genome-wide Complex Trait Analysis mtCOJO utility^59^. Output summary statistics from conditioned CanUD was then used to re-test the MR relationship between CanUD and lung cancer.

### Transcriptome-wide association study

Transcriptome-wide association studies (TWAS) and FUSION^60^ software were employed to use variant–gene expression associations to enrich GWAS variant findings for genes involved with CanUD. The TWAS models were trained using prior published evidence for gene expression from adult brain cortex^33^ (1,695 samples; 14,750 models) and fetal brain frontal cortex^34^ (201 samples; 3,784 genes), with each gene having estimated positive *cis*-heritability at nominal *P* < 0.01 and the corresponding predictive model achieving five-fold cross-validation *R*^2^ > 0.01 at a nominal *P* < 0.01. Using a weighted burden test^60^, we generated a Wald-type *Z* score for each gene–trait association, with transcriptome-wide significance defined at *P* < 2.5 × 10^−^^6^, the Bonferroni-corrected significance level across 20,000 tests. To ensure proper alignment to the genetic ancestry of the eQTL and GWAS cohorts, we use a reference panel from EUR individuals in 1KG^61^. The TWAS samples did not include any ascertainment for CanUD in the brain tissue used for analysis.

For every transcriptome-wide significant gene–trait association, we conducted a permutation test by shuffling the SNP-gene weights in the prediction model 10,000 times^60,62^. This permutation generates a null distribution to compare to the original TWAS *Z* score to quantify the significance of the expression–trait associations conditional on the SNP–trait effects at the locus^60^. For genes that passed both transcriptome-wide significance and the permutation test at *P* < 0.05 within 1 Mb of another significant gene, we conducted probabilistic gene-level fine-mapping using FOCUS to estimate 90% credible sets of genes that explain the trait association signal at a locus^63^. We conducted FOCUS fine-mapping across genes detected by models trained in either adult or fetal brain tissue.

### Partitioned SNP-based heritability estimation

To assess differences in enrichment of SNP-based trait heritability in the regions around eQTLs of adult and fetal expression, we employed stratified LDSC^61^. Genes with at least one significant eQTL were designated ‘eGenes’. We generated LD score annotations for 500-bp windows around lead eQTLs of eGenes from Genotype-Tissue Expression brain cortex (*n* = 205) and fetal brain frontal cortex (*n* = 201). We used Genotype-Tissue Expression to ensure similar sample sizes. We define the enrichment of SNP-based heritability as the proportion of heritability explained by a set of SNPs in the annotation divided by the proportion of all SNPs included in the annotation.

### gSEM

gSEM^64^ was used to perform EFA and CFA of CanUD and 14 additional traits of interest that were genetically correlated. For EFA, factor structures composed of one to ten factors were examined. EFA model fit was evaluated by the amount of cumulative variance explained by the overall factor structure, the SS loadings (SS loading ≥1) for each included factor and the proportion of explained variance accounted for by each of the individual factors (that is, ≥10%). Traits with EFA factor loadings ≥0.20 were evaluated for optimal CFA model fit determined by conventional fit indices^64^. CFA models were estimated using diagonally weighted least squares estimation and a smoothed genetic covariance matrix. The 1KG phase 3 EUR reference panel was used for LD calculation^47^.

### Multi-trait analysis of GWAS

We applied the MTAG method^65^ for the joint analysis of the genome-wide association statistics of CanUD (EUR meta-analysis from the present study), AUD (*n* = 167,721)^66^ and nicotine dependence (based on the FTND; *n* = 58,000)^67^. First, SNPs that were duplicated, had MAF ≤0.01 or had strand ambiguity were removed from the GWAS datasets. Of the 14,768,834 SNPs available from the GWAS meta-analysis of CanUD, 5,894,946 SNPs remained for the MTAG analysis after quality control. After the MTAG analysis with AUD and nicotine dependence, 3,540,940 SNPs remained. Significant variants were defined at *P* < 5 × 10^−8^.

### Reporting summary

Further information on research design is available in the Nature Portfolio Reporting Summary linked to this article.

## Online content

Any methods, additional references, Nature Portfolio reporting summaries, source data, extended data, supplementary information, acknowledgements, peer review information; details of author contributions and competing interests; and statements of data and code availability are available at 10.1038/s41588-023-01563-z.

### Supplementary information

Supplementary Information Supplementary Tables 1–16 and Supplementary Figs. 1–7.

Reporting Summary

## Data Availability

All MVP summary statistics are made available through dbGAP request under accession phs001672.v7.p1. Meta-analysis summary statistics are available through the Gelernter lab website: https://medicine.yale.edu/lab/gelernter/. Meta-analysis data will also be made available through the Complex Trait Genetics Virtual Lab: https://vl.genoma.io/. Data for TWAS models used are available as follows: TWAS models from Gandal et al 2018: https://gandallab.org/lab_resources#:~:text=Gene%2Dlevel%20TWAS%20weights%C2%A0 eQTLs from GTEx 2020: https://storage.googleapis.com/gtex_analysis_v8/single_tissue_qtl_data/GTEx_Analysis_v8_eQTL.tar eQTLs from Walker et al 2019: https://www.cell.com/cms/10.1016/j.cell.2019.09.021/attachment/a2b04323-f963-4714-8f6b-81bc24e5bed1/mmc1.xlsx.

## Code availability

Code for software and packages used in this analysis are all publicly available through the citations for each method as introduced.

## References

[1] Martins SS (2021). Racial and ethnic differences in cannabis use following legalization in US states with medical cannabis laws. JAMA Netw. Open.

[2] Cerdá M (2020). Association between recreational marijuana legalization in the United States and changes in marijuana use and cannabis use disorder from 2008 to 2016. JAMA Psychiatry.

[3] Aldington S (2008). Cannabis use and risk of lung cancer: a case-control study. Eur. Respir. J..

[4] Volkow ND (2016). Effects of cannabis use on human behavior, including cognition, motivation, and psychosis: a review. JAMA Psychiatry.

[5] Volkow, N.D. Substance use disorders in schizophrenia—clinical implications of comorbidity. *Schizophr. Bull.* **35** 469–472 (2009). 10.1093/schbul/sbp016 PMC2669586 19325163

[6] Martin JL, Gadegbeku B, Wu D, Viallon V, Laumon B (2017). Cannabis, alcohol and fatal road accidents. PLoS ONE.

[7] Demontis D (2019). Genome-wide association study implicates *CHRNA2* in cannabis use disorder. Nat. Neurosci..

[8] Bybjerg-Grauholm, J. et al. The iPSYCH2015 case-cohort sample: updated directions for unravelling genetic and environmental architectures of severe mental disorders. Preprint at *medRxiv*10.1101/2020.11.30.20237768 (2020). 10.1038/mp.2017.196 PMC5754466 28924187

[9] Boutin NT (2022). The evolution of a large biobank at Mass General Brigham. J. Personalized Med..

[10] Johnson EC (2020). A large-scale genome-wide association study meta-analysis of cannabis use disorder. Lancet Psychiatry.

[11] Gaziano JM (2016). Million Veteran Program: a mega-biobank to study genetic influences on health and disease. J. Clin. Epidemiol..

[12] Luo, Y. et al. Estimating heritability and its enrichment in tissue-specific gene sets in admixed populations. *Hum. Mol. Genet.*10.1093/hmg/ddab130 (2021). 10.1093/hmg/ddab130 PMC8330913 33987664

[13] Agrawal A (2011). A genome-wide association study of DSM-IV cannabis dependence. Addict. Biol..

[14] Minica CC (2015). Heritability, SNP- and gene-based analyses of cannabis use initiation and age at onset. Behav. Genet..

[15] Agrawal A (2014). DSM-5 cannabis use disorder: a phenotypic and genomic perspective. Drug Alcohol Depend..

[16] Verweij KJ (2013). The genetic aetiology of cannabis use initiation: a meta-analysis of genome-wide association studies and a SNP-based heritability estimation. Addict. Biol..

[17] Sherva R (2016). Genome-wide association study of cannabis dependence severity, novel risk variants, and shared genetic risks. JAMA Psychiatry.

[18] Pasman JA (2018). GWAS of lifetime cannabis use reveals new risk loci, genetic overlap with psychiatric traits, and a causal influence of schizophrenia. Nat. Neurosci..

[19] Brown BC, Ye CJ, Price AL, Zaitlen N, Asian Genetic Epidemiology Network Type 2 Diabetes Consortium (2016). Transethnic genetic-correlation estimates from summary statistics. Am. J. Hum. Genet..

[20] Saunders GRB (2022). Genetic diversity fuels gene discovery for tobacco and alcohol use. Nature.

[21] Connor, J.P. et al. Cannabis use and cannabis use disorder. *Nat. Rev. Dis. Primers*10.1038/s41572-021-00247-4 (2021). 10.1038/s41572-021-00247-4 PMC8655458 33627670

[22] Levey DF (2020). Reproducible genetic risk loci for anxiety: results from approximately 200,000 participants in the Million Veteran Program. Am. J. Psychiatry.

[23] Levey DF (2021). Bi-ancestral depression GWAS in the Million Veteran Program and meta-analysis in >1.2 million individuals highlight new therapeutic directions. Nat. Neurosci..

[24] Thorgeirsson TE (2008). A variant associated with nicotine dependence, lung cancer and peripheral arterial disease. Nature.

[25] D’Souza, D.C. et al. Consensus paper of the WFSBP task force on cannabis, cannabinoids and psychosis. *World J. Biol. Psychiatry*10.1080/15622975.2022.2038797 (2022). 10.1080/15622975.2022.2038797 35315315

[26] Gelernter J, Polimanti R (2021). Genetics of substance use disorders in the era of big data. Nat. Rev. Genet..

[27] Johnston KJA (2019). Genome-wide association study of multisite chronic pain in UK Biobank. PLoS Genet..

[28] Fisher E (2021). Cannabinoids, cannabis, and cannabis-based medicine for pain management: a systematic review of randomised controlled trials. Pain.

[29] Stockings E (2018). Cannabis and cannabinoids for the treatment of people with chronic noncancer pain conditions: a systematic review and meta-analysis of controlled and observational studies. Pain.

[30] Jayawardana S (2021). Global consumption of prescription opioid analgesics between 2009-2019: a country-level observational study. EClinicalMedicine.

[31] Nugent SM (2017). The effects of cannabis among adults with chronic pain and an overview of general harms: a systematic review. Ann. Intern. Med..

[32] *Smoking and Health: Report of the Advisory Committee to the Surgeon General of the Public Health Service* (US Department of Health, Education, and Welfare, Public Health Service, 1964).

[33] Gandal, M.J. et al. Transcriptome-wide isoform-level dysregulation in ASD, schizophrenia, and bipolar disorder. *Science* **362** (2018). 10.1126/science.aat8127 PMC6443102 30545856

[34] Walker RL (2019). Genetic control of expression and splicing in developing human brain informs disease mechanisms. Cell.

[35] Lentini JM, Alsaif HS, Faqeih E, Alkuraya FS, Fu D (2020). DALRD3 encodes a protein mutated in epileptic encephalopathy that targets arginine tRNAs for 3-methylcytosine modification. Nat. Commun..

[36] Laugel V (2010). Mutation update for the *CSB/ERCC6* and *CSA/ERCC8* genes involved in Cockayne syndrome. Hum. Mutat..

[37] Moslehi R (2020). Integrative genomic analysis implicates *ERCC6* and its interaction with *ERCC8* in susceptibility to breast cancer. Sci. Rep..

[38] Jing JJ (2017). Epistatic SNP interaction of *ERCC6* with *ERCC8* and their joint protein expression contribute to gastric cancer/atrophic gastritis risk. Oncotarget.

[39] Ronchetti D (2018). A compendium of long non-coding RNAs transcriptional fingerprint in multiple myeloma. Sci. Rep..

[40] McCrory EJ, Mayes L (2015). Understanding addiction as a developmental disorder: an argument for a developmentally informed multilevel approach. Curr. Addict. Rep..

[41] Gunn JK (2016). Prenatal exposure to cannabis and maternal and child health outcomes: a systematic review and meta-analysis. BMJ Open.

[42] Eyles DW (2021). How do established developmental risk-factors for schizophrenia change the way the brain develops?. Transl. Psychiatry.

[43] Gulsuner S (2013). Spatial and temporal mapping of de novo mutations in schizophrenia to a fetal prefrontal cortical network. Cell.

[44] Susser ES, Lin SP (1992). Schizophrenia after prenatal exposure to the Dutch Hunger Winter of 1944–1945. Arch. Gen. Psychiatry.

[45] Hatoum, A.S. et al. The addiction risk factor: a unitary genetic vulnerability characterizes substance use disorders and their associations with common correlates. *Neuropsychopharmacology*10.1038/s41386-021-01209-w (2021). 10.1038/s41386-021-01209-w PMC9372072 34750568

[46] Levey DF (2019). Genetic associations with suicide attempt severity and genetic overlap with major depression. Transl. Psychiatry.

[47] 1000 Genomes Project Consortium (2015). A global reference for human genetic variation. Nature.

[48] Loh PR (2016). Reference-based phasing using the Haplotype Reference Consortium panel. Nat. Genet..

[49] Howie B, Fuchsberger C, Stephens M, Marchini J, Abecasis GR (2012). Fast and accurate genotype imputation in genome-wide association studies through pre-phasing. Nat. Genet..

[50] Iglesias AI (2017). Haplotype reference consortium panel: practical implications of imputations with large reference panels. Hum. Mutat..

[51] Karlson EW, Boutin NT, Hoffnagle AG, Allen NL (2016). Building the partners healthcare biobank at partners personalized medicine: informed consent, return of research results, recruitment lessons and operational considerations. J. Pers. Med.

[52] Willer CJ, Li Y, Abecasis GR (2010). METAL: fast and efficient meta-analysis of genomewide association scans. Bioinformatics.

[53] Magi R (2017). Trans-ethnic meta-regression of genome-wide association studies accounting for ancestry increases power for discovery and improves fine-mapping resolution. Hum. Mol. Genet..

[54] Hasin DS (2015). Prevalence of marijuana use disorders in the United States between 2001–2002 and 2012–2013. JAMA Psychiatry.

[55] Bulik-Sullivan BK (2015). LD Score regression distinguishes confounding from polygenicity in genome-wide association studies. Nat. Genet..

[56] Lundberg M, Campos A, Renteria M, Ngo T, Partida GC (2019). Dissecting the genetic architecture of chronic pain using CTG-VL: complex-traits genetics virtual lab. Behav. Genet..

[57] Bulik-Sullivan B (2015). An atlas of genetic correlations across human diseases and traits. Nat. Genet..

[58] Hemani G (2018). The MR-Base platform supports systematic causal inference across the human phenome. Elife.

[59] Zhu Z (2018). Causal associations between risk factors and common diseases inferred from GWAS summary data. Nat. Commun..

[60] Gusev A (2016). Integrative approaches for large-scale transcriptome-wide association studies. Nat. Genet..

[61] Finucane HK (2015). Partitioning heritability by functional annotation using genome-wide association summary statistics. Nat. Genet..

[62] Bhattacharya, A. et al. Isoform-level transcriptome-wide association uncovers extensive novel genetic risk mechanisms for neuropsychiatric disorders in the human brain. Preprint at *medRxiv*10.1101/2022.08.23.22279134 (2022). 10.1038/s41588-023-01560-2 PMC10703692 38036788

[63] Mancuso N (2019). Probabilistic fine-mapping of transcriptome-wide association studies. Nat. Genet..

[64] Grotzinger AD (2019). Genomic structural equation modelling provides insights into the multivariate genetic architecture of complex traits. Nat. Hum. Behav..

[65] Turley P (2018). Multi-trait analysis of genome-wide association summary statistics using MTAG. Nat. Genet..

[66] Kranzler HR (2019). Genome-wide association study of alcohol consumption and use disorder in 274,424 individuals from multiple populations. Nat. Commun..

[67] Quach BC (2020). Expanding the genetic architecture of nicotine dependence and its shared genetics with multiple traits. Nat. Commun..

