# Supplementary material for: Multi-ancestry genome-wide association study of cannabis use disorder yields insight into disease biology and public health implications
Source: Nat Genet. 2023 Nov 20;55(12):2094–103. doi: 10.1038/s41588-023-01563-z (PMC10703690; doi:10.1038/s41588-023-01563-z)

nature genetics

Article

https://doi.org/10.1038/s41588-023-01563-z

Multi-ancestry genome-wide association study of cannabis use disorder yields insight into disease biology and public health implications

In the format provided by the authors and unedited

Supplementary Information

Contents

Supplementary Table 1. Within trait r_G

Supplementary Table 2. ICD-9 codes used in the definition of MVP cases

Supplementary Table 3. ICD-10 codes used in the definition of MVP cases

Supplementary Table 4. TWAS

Supplementary Table 5. MR analysis of CanUD on Multisite Chronic Pain

Supplementary Table 6. MR analysis of CanUD on Physical Activity

Supplementary Table 7. MR analysis of CanUD on Schizophrenia

Supplementary Table 8. MR analysis of CanUD on Schizophrenia using 1x10-5 threshold

Supplementary Table 9. MR analysis of Multisite Chronic Pain on CanUD using 1x10-5 threshold

Supplementary Table 10. MR analysis of Physical Activity

Supplementary Table 11. MR analysis of Schizophrenia on CanUD using 1x10-5 threshold

Supplementary Table 12. MR analysis of CanUD on Lung cancer using 1x10-5 threshold

Supplementary Table 13. MR analysis of CanUD conditioned on smoking initiation

Supplementary Table 14. MTAG association results

Supplementary Table 15. MTAG effect size comparison

Supplementary Table 16. Extended Demographics

Supplementary Figure 1. Mendelian Randomization

Supplementary Figure 2. Cross Trait rG

Supplementary Figure 3. Cross Ancestry Genetic Correlation

Supplementary Figure 4. Pathway enrichment

Supplementary Figure 6. GWAS Catalog Reported Gene Enrichment

Supplementary Figure 7. Sex Stratified GWAS Within the MVP

Supplementary table 1. Within trait r_G. Tests were 2-sided. P-values are uncorrected.

Trait 1	Trait 2	rG	se	z	p
MVP_Definition1	MVP_Definition2	0.99	0.0076	130.58	0.00E+00
MVP_Definition1	PGC_deCODE_CUD	0.77	0.0661	11.64	2.67E-31
MVP_Definition2	PGC_deCODE_CUD	0.75	0.063	11.93	7.80E-33
MVP_Definition1	iPSYCH2	0.70	0.0817	8.57	9.97E-18
MVP_Definition2	iPSYCH2	0.66	0.0807	8.14	3.96E-16
PGC_deCODE_CUD	iPSYCH2	0.87	0.102	8.51	1.80E-17

Supplementary Table 2. ICD-9 codes used in the definition of MVP cases.

ICD Code	ICD Code Description	Code Type
304.3	MARIJUANA DEPENDENCE UNSPECIFIED	ICD-9
304.3	CANNABIS DEPENDENCE UNSPECIFIED	ICD-9
304.309	OTHER CANNABIS DEPENDENCE UNSPECIFIED	ICD-9
304.31	CANNABIS DEPENDENCE, CONTINUOUS USE	ICD-9
304.31	MARIJUANA DEPENDENCE CONTINUOUS	ICD-9
304.319	OTHER CANNABIS DEPENDENCE CONTINUOUS	ICD-9
304.32	CANNABIS DEPENDENCE, EPISODIC USE	ICD-9
304.32	MARIJUANA DEPENDENCE EPISODIC	ICD-9
304.329	OTHER CANNABIS DEPENDENCE EPISODIC	ICD-9
304.33	CANNABIS DEPENDENCE, IN REMISSION	ICD-9
304.33	MARIJUANA DEPENDENCE IN REMISSION	ICD-9
304.339	OTHER CANNABIS DEPENDENCE IN REMISSION	ICD-9
304.39	OTHER CANNABIS DEPEND, NEC	ICD-9
305.2	CANNABIS ABUSE UNSPECIFIED	ICD-9
305.2	MARIJUANA ABUSE UNSPECIFIED	ICD-9
305.209	OTHER CANNABIS ABUSE UNSPECIFIED	ICD-9
305.21	CANNABIS ABUSE, CONTINUOUS USE	ICD-9
305.21	MARIJUANA ABUSE CONTINUOUS	ICD-9
305.219	OTHER CANNABIS ABUSE CONTINUOUS	ICD-9
305.22	CANNABIS ABUSE, EPISODIC USE	ICD-9
305.22	MARIJUANA ABUSE EPISODIC	ICD-9
305.229	OTHER CANNABIS ABUSE EPISODIC	ICD-9
305.23	CANNABIS ABUSE, IN REMISSION	ICD-9
305.23	MARIJUANA ABUSE IN REMISSION	ICD-9
305.239	OTHER CANNABIS ABUSE IN REMISSION	ICD-9
305.29	OTHER CANNABIS USE, NEC	ICD-9

Supplementary Table 3. ICD-10 codes used in the definition of MVP cases.

ICD Code	ICD Code Description	Code Type
F12.10	Cannabis abuse, uncomplicated	ICD-10
F12.11	Cannabis abuse, in remission	ICD-10
F12.120	Cannabis abuse with intoxication, uncomplicated	ICD-10
F12.121	Cannabis abuse with intoxication delirium	ICD-10
F12.122	Cannabis abuse with intoxication with perceptual disturbance	ICD-10
F12.129	Cannabis abuse with intoxication, unspecified	ICD-10
F12.150	Cannabis abuse with psychotic disorder with delusions	ICD-10
F12.151	Cannabis abuse with psychotic disorder with hallucinations	ICD-10
F12.159	Cannabis abuse with psychotic disorder, unspecified	ICD-10
F12.180	Cannabis abuse with cannabis-induced anxiety disorder	ICD-10
F12.188	Cannabis abuse with other cannabis-induced disorder	ICD-10
F12.19	Cannabis abuse with unspecified cannabis-induced disorder	ICD-10
F12.20	Cannabis dependence, uncomplicated	ICD-10
F12.21	Cannabis dependence, in remission	ICD-10
F12.220	Cannabis dependence with intoxication, uncomplicated	ICD-10
F12.221	Cannabis dependence with intoxication delirium	ICD-10
F12.222	Cannabis dependence with intoxication with perceptual disturbance	ICD-10
F12.229	Cannabis dependence with intoxication, unspecified	ICD-10
F12.23	Cannabis dependence with withdrawal	ICD-10
F12.250	Cannabis dependence with psychotic disorder with delusions	ICD-10
F12.251	Cannabis dependence with psychotic disorder with hallucinations	ICD-10
F12.259	Cannabis dependence with psychotic disorder, unspecified	ICD-10
F12.280	Cannabis dependence with cannabis-induced anxiety disorder	ICD-10
F12.288	Cannabis dependence with other cannabis-induced disorder	ICD-10
F12.29	Cannabis dependence with unspecified cannabis-induced disorder	ICD-10
F12.90	Cannabis use, unspecified, uncomplicated	ICD-10
F12.920	Cannabis use, unspecified with intoxication, uncomplicated	ICD-10
F12.921	Cannabis use, unspecified with intoxication delirium	ICD-10
F12.922	Cannabis use, unspecified with intoxication with perceptual disturbance	ICD-10
F12.929	Cannabis use, unspecified with intoxication, unspecified	ICD-10
F12.93	Cannabis use, unspecified with withdrawal	ICD-10
F12.950	Cannabis use, unspecified with psychotic disorder with delusions	ICD-10
F12.951	Cannabis use, unspecified with psychotic disorder with hallucinations	ICD-10
F12.959	Cannabis use, unspecified with psychotic disorder, unspecified	ICD-10
F12.980	Cannabis use, unspecified with anxiety disorder	ICD-10
F12.988	Cannabis use, unspecified with other cannabis-induced disorder	ICD-10
F12.99	Cannabis use, unspecified with unspecified cannabis-induced disorder	ICD-10

Supplementary Table 4. TWAS. 4 distinct GWAS-significant loci and 38 novel genetic loci across adult and fetal brain cortex associated with cannabis use, with limited overlap across the two tissues.

CHR	Gene	eQTL Dataset	TWAS Z-score	TWAS P-value	Permutation P-value
16	PHLPP2	Adult brain cortex (Gandal 2018)	-7.34	2.17E-13	9.99E-04
3	DALRD3	Adult brain cortex (Gandal 2018)	-6.15	7.51E-10	4.91E-02
3	DALRD3	Fetal brain cortex (Walker 2019)	-7.08	1.45E-12	9.99E-04
11	RP11-629G13.1	Adult brain cortex (Gandal 2018)	-5.20	1.98E-07	1.50E-03
5	ERCC8	Fetal brain cortex (Walker 2019)	5.16	2.44E-07	1.35E-02
2	LY75	Adult brain cortex (Gandal 2018)	6.20	5.58E-10	7.99E-03
3	CRYBG3	Adult brain cortex (Gandal 2018)	-4.94	7.99E-07	3.19E-02
4	RP11-700J17.2	Fetal brain cortex (Walker 2019)	-4.90	9.77E-07	1.10E-03
5	RP11-343L5.2	Adult brain cortex (Gandal 2018)	12.26	1.43E-34	2.40E-03
6	RP1-8B1.4	Adult brain cortex (Gandal 2018)	-5.57	2.52E-08	9.99E-04
6	PKMP3	Adult brain cortex (Gandal 2018)	6.38	1.75E-10	9.99E-04
6	RP11-350J20.9	Adult brain cortex (Gandal 2018)	8.22	2.04E-16	1.30E-02
7	HIBADH	Adult brain cortex (Gandal 2018)	-4.88	1.07E-06	4.40E-03
7	CLEC5A	Adult brain cortex (Gandal 2018)	7.91	2.47E-15	2.41E-02
8	LNCOC1	Adult brain cortex (Gandal 2018)	5.44	5.44E-08	9.99E-04
9	RP11-498P14.5	Adult brain cortex (Gandal 2018)	4.85	1.21E-06	2.35E-02
10	BMS1P4	Adult brain cortex (Gandal 2018)	5.44	5.28E-08	9.99E-04
12	TAS2R64P	Adult brain cortex (Gandal 2018)	9.49	2.23E-21	3.46E-02
12	LINC02361	Adult brain cortex (Gandal 2018)	-9.19	4.06E-20	1.97E-02
15	RYR3-DT	Adult brain cortex (Gandal 2018)	-5.52	3.34E-08	1.50E-03
15	SPG21	Adult brain cortex (Gandal 2018)	10.74	6.43E-27	9.99E-04
16	RP11-473I1.9	Adult brain cortex (Gandal 2018)	-4.97	6.77E-07	3.04E-02
17	TMEM220	Adult brain cortex (Gandal 2018)	-6.20	5.77E-10	9.99E-04
17	USP36	Adult brain cortex (Gandal 2018)	8.30	9.99E-17	2.70E-03
1	SRGAP2C	Fetal brain cortex (Walker 2019)	-10.12	4.61E-24	9.99E-04
14	LGALS3	Adult brain cortex (Gandal 2018)	4.81	1.48E-06	9.99E-04
11	PPP1R14B	Adult brain cortex (Gandal 2018)	4.82	1.43E-06	1.10E-03
21	PIGP	Fetal brain cortex (Walker 2019)	-5.94	2.78E-09	2.89E-02
5	TGFBI	Adult brain cortex (Gandal 2018)	-4.76	1.91E-06	8.19E-03
1	SDCCAG8	Adult brain cortex (Gandal 2018)	4.94	7.66E-07	9.69E-03
8	RP11-16E18.3	Adult brain cortex (Gandal 2018)	19.73	1.2E-86	1.20E-03
2	PLCL1	Adult brain cortex (Gandal 2018)	4.76	1.95E-06	9.99E-04
19	WDR18	Fetal brain cortex (Walker 2019)	5.38	7.58E-08	9.99E-04
1	RP11-54O7.18	Adult brain cortex (Gandal 2018)	6.77	1.25E-11	2.66E-02
12	ACSS3	Fetal brain cortex (Walker 2019)	-7.72	1.15E-14	9.99E-04
8	ASAH1	Fetal brain cortex (Walker 2019)	-5.62	1.91E-08	9.99E-04
16	YPEL3	Fetal brain cortex (Walker 2019)	-5.60	2.16E-08	4.90E-02
1	RP11-338N10.2	Fetal brain cortex (Walker 2019)	5.26	1.44E-07	1.60E-03
1	PBXIP1	Adult brain cortex (Gandal 2018)	5.69	1.24E-08	9.99E-04
11	KRTAP5-AS1	Fetal brain cortex (Walker 2019)	5.24	1.6E-07	9.99E-04

2	RP11-482H16.1	Adult brain cortex (Gandal 2018)	-8.69	3.77E-18	9.99E-04
1	ST3GAL3	Fetal brain cortex (Walker 2019)	5.01	5.32E-07	6.99E-03
6	PTMAP1	Fetal brain cortex (Walker 2019)	-4.73	2.27E-06	9.99E-04

Supplementary Table 5. MR analysis of CanUD on Multisite Chronic Pain using $5x10^{-8}$ threshold for instrument selection. Tests are two sided. Values shown are uncorrected.

**Cannabis use disorder --> Multisite chronic pain (P_T=$5x10^{-8}$)**				
**(1) test for heterogeneity among genetic instrument**				
method	Q	Q_df	Q_pval	
MR Egger	60.69	17	$8.08x10^{-7}$	
Inverse variance weighted	101.18	18	$1.34x10^{-13}$	
**(2) test for evidence of horizontal pleiotropy among genetic instrument**				
egger_intercept	se	pval		
0.013	0.004	0.004		
**(3) retest heterogeneity after outlier removal**				
method	Q	Q_df	Q_pval	
MR Egger	15.49	10	0.115	
Inverse variance weighted	18.36	11	0.074	
**(4) retest horizontal pleiotropy after outlier removal**				
egger_intercept	se	pval		
0.004	0.003	0.203		
**(5) MR-RAPS overdispersion**				
tau.hat	tau.se	pval		
$9.41x10^{-7}$	$4.60x10^{-6}$	0.838		
**(6) test causal effect of exposure on outcome**				
method	nsnp	b	se	pval
MR Egger	12	-0.042	0.047	0.390
Weighted Median	12	0.028	0.017	0.098
IVW	12	0.019	0.015	0.229
Simple Mode	12	0.029	0.030	0.362
Weighted Mode	12	0.019	0.022	0.421
Robust Adjusted Profile Score (RAPS)	12	0.018	0.016	0.259

Supplementary Table 6. MR analysis of CanUD on Physical Activity (none) using $1x10^{-8}$ threshold for instrument selection. Tests are two sided. Values shown are uncorrected.

**Cannabis use disorder --> Physical activity (none) (P_T=$5x10^{-8}$)**				
**(1) test for heterogeneity among genetic instrument**				
method	Q	Q_df	Q_pval	
MR Egger	15.86	17	0.534	
Inverse variance weighted	22.93	18	0.193	
**(2) test for evidence of horizontal pleiotropy among genetic instrument**				
egger_intercept	se	pval		
-0.022	0.008	0.016		
**(3) retest heterogeneity after outlier removal**				
method	Q	Q_df	Q_pval	
MR Egger	9.40	13	0.742	
Inverse variance weighted	12.13	14	0.596	
**(4) retest horizontal pleiotropy after outlier removal**				
egger_intercept	se	pval		
-0.015	0.009	0.122		
**(5) MR-RAPS overdispersion**				
tau.hat	tau.se	pval		
$7.13x10^{-9}$	$5.36x10^{-5}$	0.999		
**(6) test causal effect of exposure on outcome**				
method	nsnp	b	se	pval
MR Egger	15	0.116	0.140	0.419
Weighted Median	15	0.123	0.063	0.050
IVW	15	0.103	0.044	0.018
Simple Mode	15	0.159	0.106	0.154
Weighted Mode	15	0.147	0.099	0.159
Robust Adjusted Profile Score (RAPS)	15	0.114	0.046	0.014

Supplementary Table 7. MR analysis of CanUD on Schizophrenia using 1x10^{-8} threshold for instrument selection. Tests are two sided. Values shown are uncorrected.

**Cannabis use disorder --> Schizophrenia (P_T=5x10^{-8})**				
**(1) test for heterogeneity among genetic instrument**				
method	Q	Q_df	Q_pval	
MR Egger	2.338	2	0.3106	
Inverse variance weighted	2.453	3	0.4839	
**(2) test for evidence of horizontal pleiotropy among genetic instrument**				
egger_intercept	se	pval		
0.0263	0.0843	0.7843		
**(3) retest heterogeneity after outlier removal**				
method	Q	Q_df	Q_pval	
MR Egger	-	-	-	
Inverse variance weighted	-	-	-	
**(4) retest horizontal pleiotropy after outlier removal**				
egger_intercept	se	pval		
-	-	-		
**(5) MR-RAPS overdispersion**				
tau.hat	tau.se	pval		
7.804x10^{-9}	4.957x10^{-5}	0.999		
**(6) test causal effect of exposure on outcome**				
method	nsnp	b	se	pval
MR Egger	4	0.838	3.06	0.810
Weighted Median	4	0.107	0.213	0.616
IVW	4	0.115	0.159	0.469
Simple Mode	4	0.195	0.284	0.542
Weighted Mode	4	0.113	0.278	0.711
Robust Adjusted Profile Score (RAPS)	4	0.127	0.186	0.496

Supplementary Table 8. MR analysis of CanUD on Schizophrenia using $1x10^{-5}$ threshold for instrument selection. Tests are two sided. Values shown are uncorrected.

**Cannabis use disorder --> Schizophrenia (P_T=$1x10^{-5}$)**				
**(1) test for heterogeneity among genetic instrument**				
method	Q	Q_df	Q_pval	
MR Egger	51.479	29	0.0062	
Inverse variance weighted	52.56	30	0.0066	
**(2) test for evidence of horizontal pleiotropy among genetic instrument**				
egger_intercept	se	pval		
0.0072	0.0092	0.442		
**(3) retest heterogeneity after outlier removal**				
method	Q	Q_df	Q_pval	
MR Egger	16.471	27	0.943	
Inverse variance weighted	18.131	28	0.923	
**(4) retest horizontal pleiotropy after outlier removal**				
egger_intercept	se	pval		
0.0093	0.0072	0.208		
**(5) MR-RAPS overdispersion**				
tau.hat	tau.se	pval		
$9.669x10^{-9}$	$2.805x10^{-5}$	0.999		
**(6) test causal effect of exposure on outcome**				
method	nsnp	b	se	pval
MR Egger	29	0.1622	0.2743	0.5593
Weighted Median	29	0.1861	0.1002	0.0632
IVW	29	0.1797	0.0698	0.0101
Simple Mode	29	0.2243	0.1842	0.1955
Weighted Mode	29	0.2235	0.1811	0.2276
Robust Adjusted Profile Score (RAPS)	29	0.1922	0.0859	0.0251

Supplementary Table 9. MR analysis of Multisite Chronic Pain on CanUD using $1x10^{-5}$ threshold for instrument selection. Tests are two sided. Values shown are uncorrected.

**Multisite chronic pain --> Cannabis use disorder (P_T=$5x10^{-8}$)**				
**(1) test for heterogeneity among genetic instrument**				
method	Q	Q_df	Q_pval	
MR Egger	117.96	41	$2.24x10^{-9}$	
Inverse variance weighted	120.89	42	$1.44x10^{-9}$	
**(2) test for evidence of horizontal pleiotropy among genetic instrument**				
egger_intercept	se	pval		
0.014	0.013	0.319		
**(3) retest heterogeneity after outlier removal**				
method	Q	Q_df	Q_pval	
MR Egger	35.89	36	0.473	
Inverse variance weighted	36.81	37	0.478	
**(4) retest horizontal pleiotropy after outlier removal**				
egger_intercept	se	pval		
-0.009	0.009	0.342		
**(5) MR-RAPS overdispersion**				
tau.hat	tau.se	pval		
$7.09x10^{-6}$	$2.62x10^{-5}$	0.787		
**(6) test causal effect of exposure on outcome**				
method	nsnp	b	se	pval
MR Egger	38	1.03	0.603	0.046
Weighted Median	38	0.680	0.150	$6.24x10^{-6}$
IVW	38	0.460	0.110	$2.90x10^{-5}$
Simple Mode	38	0.752	0.299	0.016
Weighted Mode	38	0.742	0.281	0.012
Robust Adjusted Profile Score (RAPS)	38	0.514	0.118	$1.25x10^{-5}$

Supplementary Table 10. MR analysis of Physical Activity (none) on CanUD using $1x10^{-6}$ threshold for instrument selection. Tests are two sided. Values shown are uncorrected.

**Physical activity (none) --> Cannabis use disorder (P_T=$1x10^{-6}$)**				
**(1) test for heterogeneity among genetic instrument**				
method	Q	Q_df	Q_pval	
MR Egger	16.65	11	0.119	
Inverse variance weighted	16.78	12	0.158	
**(2) test for evidence of horizontal pleiotropy among genetic instrument**				
egger_intercept	se	Pval		
-0.023	-0.005	0.016	0.781	
**(3) retest heterogeneity after outlier removal**				
Method	Q	Q_df	Q_pval	
MR Egger	-	-	-	
Inverse variance weighted	-	-	-	
**(4) retest horizontal pleiotropy after outlier removal**				
egger_intercept	se	pval		
-	-	-		
**(5) MR-RAPS overdispersion**				
tau.hat	tau.se	pval		
$2.32x10^{-5}$	$9.73x10^{-5}$	0.812		
**(6) test causal effect of exposure on outcome**				
method	nsnp	b	se	pval
MR Egger	13	0.200	0.241	0.424
Weighted Median	13	0.261	0.084	0.002
IVW	13	0.266	0.069	$1.17x10^{-4}$
Simple Mode	13	0.241	0.136	0.101
Weighted Mode	13	0.225	0.115	0.074
Robust Adjusted Profile Score (RAPS)	13	0.271	0.078	$5.06x10^{-4}$

Supplementary Table 11. MR analysis of Schizophrenia on CanUD using $1x10^{-5}$ threshold for instrument selection. Tests are two sided. Values shown are uncorrected.

**Schizophrenia --> Cannabis use disorder (P_T=$5x10^{-8}$)**				
**(1) test for heterogeneity among genetic instrument**				
method	Q	Q_df	Q_pval	
MR Egger	53.809	119	1	
Inverse variance weighted	52.323	120	1	
**(2) test for evidence of horizontal pleiotropy among genetic instrument**				
egger_intercept	se	pval		
0.003	0.004	0.475		
**(3) retest heterogeneity after outlier removal**				
method	Q	Q_df	Q_pval	
MR Egger	-	-	-	
Inverse variance weighted	-	-	-	
**(4) retest horizontal pleiotropy after outlier removal**				
egger_intercept	se	pval		
-	-	-		
**(5) MR-RAPS overdispersion**				
tau.hat	tau.se	pval		
$8.87x10^{-9}$	$1.68x10^{-5}$	0.999		
**(6) test causal effect of exposure on outcome**				
method	nsnp	b	se	pval
MR Egger	121	0.0546	0.1592	0.7324
Weighted Median	121	0.1601	0.0534	0.0031
IVW	121	0.1653	0.0388	$2.07x10^{-5}$
Simple Mode	121	0.1243	0.1434	0.3877
Weighted Mode	121	0.1243	0.1372	0.3669
Robust Adjusted Profile Score (RAPS)	121	0.1816	0.0427	$2.07x10^{-5}$

Supplementary Table 12. MR analysis of CanUD on Lung cancer using 1x10^{-5} threshold for instrument selection. Tests are two sided. Values shown are uncorrected.

**Cannabis use disorder --> Lung cancer (P_T=10^{-5})**				
**(1) test for heterogeneity among genetic instrument**				
method	Q	Q_df	Q_pval	
MR Egger	132.67	90	0.002	
Inverse variance weighted	133.70	91	0.002	
**(2) test for evidence of horizontal pleiotropy among genetic instrument**				
egger_intercept	se	pval		
0.009	0.011	0.40		
**(3) retest heterogeneity after outlier removal**				
method	Q	Q_df	Q_pval	
MR Egger	129.76	89	0.003	
Inverse variance weighted	131.07	90	0.003	
**(4) retest horizontal pleiotropy after outlier removal**				
egger_intercept	se	pval		
0.010	0.011	0.35		
**(5) MR-RAPS overdispersion**				
tau2.hat	tau2.se	beta.hat	beta.se	beta.pval
5.0x10^{-4}	3.7x10^{-4}	0.207	0.064	0.001
**(6) test causal effect of exposure on outcome**				
method	nsnp	b	se	pval
MR Egger	92	0.06	0.16	0.71
Weighted Median	92	0.25	0.09	0.006
IVW	92	0.18	0.07	0.006
Simple Mode	92	0.29	0.22	0.21
Weighted Mode	92	0.25	0.18	0.18
Robust Adjusted Profile Score (RAPS)	92	0.206	0.054	0.0001

Supplementary Table 13. MR analysis of CanUD conditioned on smoking initiation from GSCAN2[34] on Lung cancer using $1x10^{-5}$ threshold for instrument selection. Tests are two sided. Values shown are uncorrected.

**Cannabis use disorder --> Lung cancer ($P_T=10^{-5}$)**				
**(1) test for heterogeneity among genetic instrument**				
method	Q	Q_df	Q_pval	
MR Egger	130.07	83	$7.4x10^{-4}$	
Inverse variance weighted	134.20	84	$4.1x10^{-4}$	
**(2) test for evidence of horizontal pleiotropy among genetic instrument**				
egger_intercept	se	pval		
-0.025	0.015	0.11		
**(3) retest heterogeneity after outlier removal**				
method	Q	Q_df	Q_pval	
MR Egger	128.46	82	$8.0x10^{-4}$	
Inverse variance weighted	131.64	83	$5.4x10^{-4}$	
**(4) retest horizontal pleiotropy after outlier removal**				
egger_intercept	se	pval		
-0.022	0.016	0.16		
**(5) MR-RAPS overdispersion**				
tau2.hat	tau2.se	beta.hat	beta.se	beta.pval
$7.9x10^{-4}$	$3.7x10^{-4}$	0.204	0.089	0.021
**(6) test causal effect of exposure on outcome**				
method	nsnp	b	se	pval
MR Egger	85	0.64	0.28	0.027
Weighted Median	85	0.23	0.11	0.032
IVW	85	0.20	0.089	0.025
Simple Mode	85	0.21	0.27	0.44
Weighted Mode	85	0.28	0.26	0.28

Robust Adjusted Profile Score (RAPS)	85	0.198	0.070	$4.6x10^{-3}$

Supplementary Table 14. MTAG association results for genomic risk loci for CanUD/ND/AUD. Tests were two-sided. P-values are uncorrected.

rsID	CHR	BP	Effect allele	Other allele	EAF	Beta	SE	P value
rs10799122	1	29168766	T	G	0.33	0.019	0.0034	4.01E-08
rs7519259	1	66434743	A	G	0.52	0.024	0.0032	1.12E-13
rs12061680	1	73778378	A	G	0.59	-0.021	0.0032	6.63E-11
rs1526480	1	91209986	T	C	0.38	-0.022	0.0033	5.18E-12
rs3897854	1	97918306	T	C	0.45	-0.017	0.0032	4.25E-08
rs472140	2	45139904	T	C	0.58	0.021	0.0032	1.44E-10
rs1594166	2	185787586	T	G	0.66	-0.019	0.0034	1.05E-08
rs1372072	3	16955259	A	G	0.36	0.019	0.0033	5.08E-09
rs12497569	3	49364830	A	G	0.56	-0.024	0.0032	4.32E-14
rs9834272	3	55967193	T	C	0.05	0.042	0.0074	2.06E-08
rs726610	3	85551403	T	C	0.64	-0.022	0.0033	2.33E-11
rs13107325	4	103188709	T	C	0.08	-0.041	0.0057	5.05E-13
rs4546329	5	60589739	T	C	0.49	0.019	0.0032	1.72E-09
rs62461186	7	77730153	A	C	0.17	0.026	0.0042	4.93E-10
rs1989903	7	114137940	A	G	0.59	-0.019	0.0032	6.31E-09
rs11778040	8	27419807	T	C	0.20	-0.023	0.0040	5.34E-09
rs864882	9	127968109	T	C	0.30	-0.024	0.0034	2.63E-12
rs1637570	10	118619529	A	G	0.72	-0.020	0.0035	8.41E-09
rs10835372	11	28643913	T	C	0.37	0.021	0.0033	1.67E-10
rs11570190	11	57560452	A	C	0.33	-0.022	0.0034	6.39E-11
rs6589386	11	113443753	T	C	0.43	-0.023	0.0032	2.04E-12
rs1944688	11	121635463	A	G	0.76	0.022	0.0037	5.42E-09
rs850254	14	57350427	A	G	0.57	-0.018	0.0032	2.58E-08
rs73403005	15	47645174	A	G	0.17	-0.024	0.0043	2.24E-08
rs4702	15	91426560	A	G	0.55	-0.018	0.0032	3.32E-08
rs72833102	17	55663552	A	G	0.15	0.025	0.0045	1.55E-08

Supplementary Table 15. Effect size comparison between the variants significant in the MTAG analysis and GWAS. The difference in effect sizes reaching the Bonferroni significant threshold (1.92E-03) are highlighted in green. Tests were two-sided.

rsid	Beta GWAS	se GWAS	pval GWAS	beta MTAG	se MTAG	pval MTAG	z score	p value
rs10799122	0.045	0.009	2.95E-07	0.019	0.003	4.01E-08	2.998	4.46E-03
rs7519259	0.050	0.008	1.83E-09	0.024	0.003	1.12E-13	3.197	2.41E-03
rs12061680	-0.044	0.008	1.09E-07	-0.021	0.003	6.63E-11	-2.776	8.47E-03
rs1526480	-0.052	0.008	5.91E-10	-0.022	0.003	5.18E-12	-3.502	8.67E-04
rs3897854	-0.036	0.009	3.63E-05	-0.017	0.003	4.25E-08	-2.150	3.95E-02
rs472140	0.040	0.009	2.02E-06	0.021	0.003	1.44E-10	2.328	2.65E-02
rs1594166	-0.041	0.009	2.15E-06	-0.019	0.003	1.05E-08	-2.528	1.63E-02
rs1372072	0.041	0.009	1.60E-06	0.019	0.003	5.08E-09	2.516	1.69E-02
rs12497569	-0.057	0.008	7.54E-12	-0.024	0.003	4.32E-14	-3.916	1.87E-04
rs9834272	0.097	0.019	1.57E-07	0.042	0.007	2.06E-08	2.986	4.62E-03
rs726610	-0.056	0.009	4.29E-11	-0.022	0.003	2.33E-11	-4.029	1.19E-04
rs13107325	-0.077	0.017	3.36E-06	-0.041	0.006	5.05E-13	-2.129	4.14E-02
rs4546329	0.045	0.008	1.01E-07	0.019	0.003	1.72E-09	3.059	3.70E-03
rs62461186	0.068	0.011	8.76E-10	0.026	0.004	4.93E-10	3.768	3.30E-04
rs1989903	-0.047	0.008	1.55E-08	-0.019	0.003	6.31E-09	-3.403	1.22E-03
rs11778040	-0.070	0.011	3.22E-11	-0.023	0.004	5.34E-09	-4.463	1.89E-05
rs864882	-0.057	0.009	3.12E-10	-0.024	0.003	2.63E-12	-3.599	6.14E-04
rs1637570	-0.050	0.009	4.79E-08	-0.020	0.004	8.41E-09	-3.205	2.35E-03
rs10835372	0.047	0.009	3.03E-08	0.021	0.003	1.67E-10	3.078	3.49E-03
rs11570190	-0.044	0.009	2.88E-07	-0.022	0.003	6.39E-11	-2.592	1.39E-02
rs6589386	-0.040	0.008	1.10E-06	-0.023	0.003	2.04E-12	-2.140	4.04E-02
rs1944688	0.047	0.010	6.22E-06	0.022	0.004	5.42E-09	2.420	2.13E-02
rs850254	-0.047	0.009	2.58E-07	-0.018	0.003	2.58E-08	-3.187	2.48E-03
rs73403005	-0.039	0.011	3.22E-04	-0.024	0.004	2.24E-08	-1.414	1.47E-01
rs4702	-0.047	0.009	1.67E-07	-0.018	0.003	3.32E-08	-3.270	1.90E-03
rs72833102	0.055	0.012	1.71E-06	0.025	0.004	1.55E-08	2.592	1.39E-02

Supplementary Table 16. Extended Demographics. Extended data where available. Specific values were not available for all cohorts based on previously published data. Age was not available in all PGC samples

	Age	Ascertainment	%F	SES?
MVP	63.8	US Military veterans using the US Department of Veterans Affairs national healthcare system	7.4%	$39,190
CATS	36	Opioid substitution clinics	40%	83% unemployed
CADD			30%	
CHDS	18-35	Birth cohort	52%	
FSCD	36.5	Treatment Centers	53%	25-60% unemployed
COGEND	37	community-based & treatment	20-49%	10% less than high school
GEDI-GSMS	9y-30y*	Rural community	47%	Rural
ADAA	41.2	treatment centers	57%	
BLTS	26 (18-38)	community twins	58%	
MCTFR	20.6	community twins	46%	
Yale-Penn	39.5		42%	
COGA		in-patient treatment centers & out-patient clinics, community comparison families	55%	
CEDAR		Various (community & treatment)	37%	
OZ-ALC	43	community-based	49%	
VTSABD	8y-30y*	cohort longitudinal	62%	
IASPSAD	41	treatment facilities	50%	35% unemployed
deCODE	66.5	Treatment Center	59.50%	

*Age range reported for longitudinal cohort.

ADAA=Alcohol Dependence in African Americans. BLTS= Brisbane Longitudinal Twin Study. CADD=Center on Antisocial Drug Dependence. CATS=Comorbidity and Trauma Study. CEDAR= Center for Education and Drug Abuse Research. CHDS=Christchurch Health and Development Study. COGA=Collaborative Study on the Genetics of Alcoholism. COGEND=Collaborative Genetic Study of Nicotine Dependence. FSCD=Family Study of Cocaine Dependence. GEDI=Gene-Environment-Development Initiative. GSMS=Great Smoky Mountains Study. IASPSAD=Irish Affected Sib-Pair Study of Alcohol Dependence. MCTFR= Minnesota Center for Twin and Family Research. OZ-ALC=Australian Alcohol and Nicotine Studies. VTSABD=Virginia Twin Studies of Adolescent Behavioral Development. deCODE = deCODE genetics

Supplementary Figure 1. Mendelian Randomization. On the left are the effect of CanUD instruments on (from top to bottom) schizophrenia, multisite chronic pain, and physical activity past 4 weeks (None). On the right are instruments from schizophrenia, multisite chronic pain, and physical activity past 4 weeks (None), respectively, on CanUD. Bilateral effect was observed for schizophrenia, while a unidirectional effect was found for multisite chronic pain on CanUD. Standard error depicted on each axis for each variant/trait relationship.

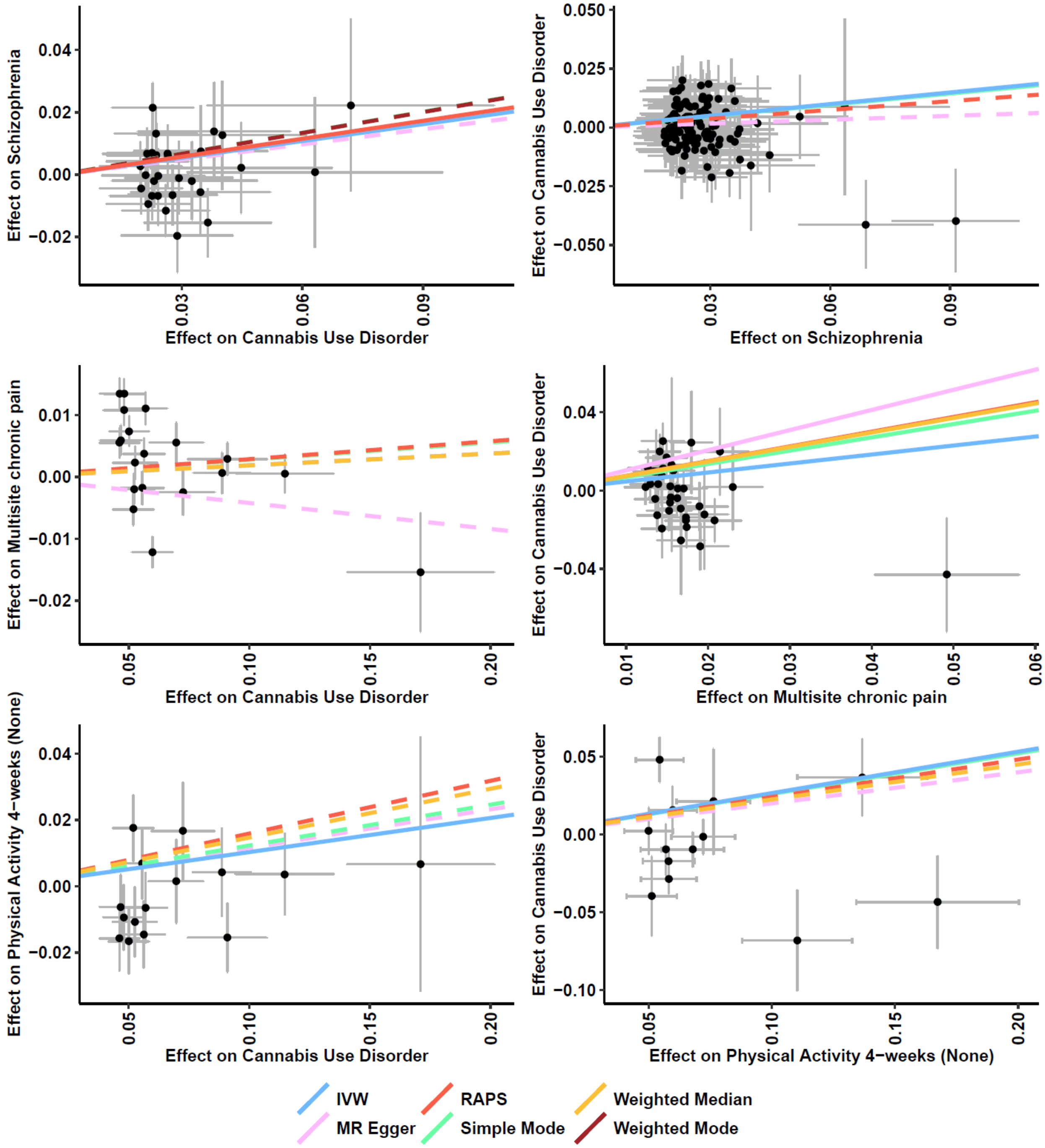

Supplementary Figure 2. Cross Trait rG. Multi-trait r_G volcano plot calculated using 1,335 traits available in CTG-Lab. Red lines indicate a threshold of absolute rG > 0.50 (x-axis) or significant p-value following correction for multiple comparisons for 1335 traits. Statistical tests were two-sided.

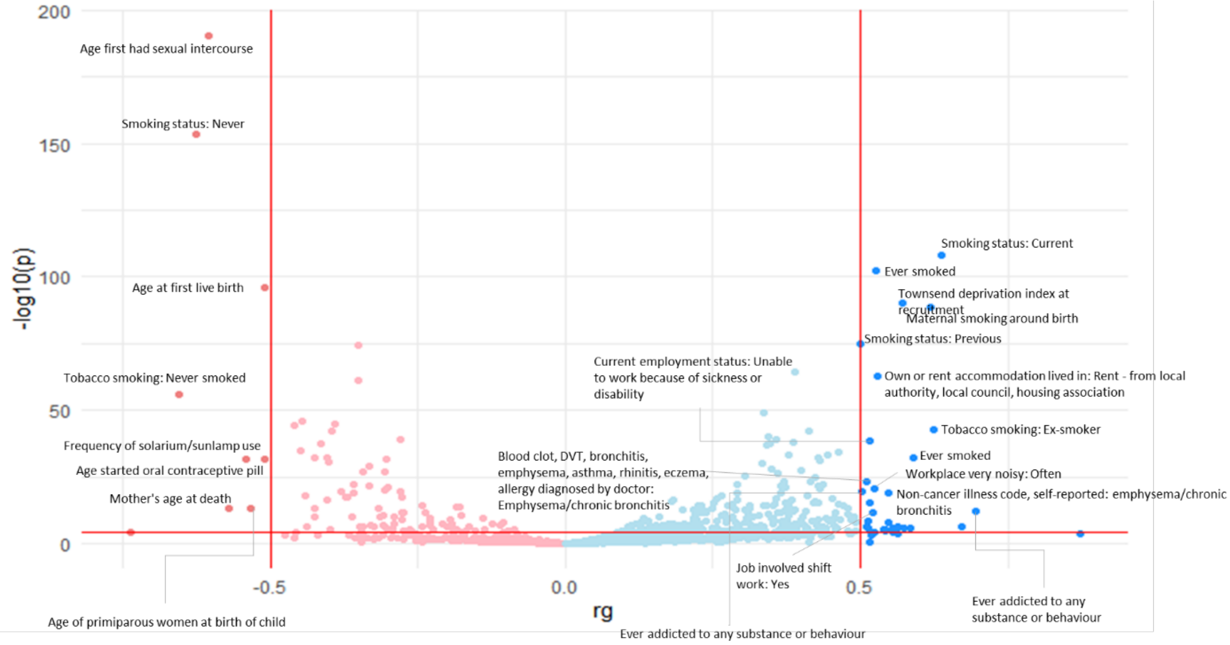

Supplementary Figure 3. Cross Ancestry Genetic Correlation. X axis (left) depicts the range of rG between 1 and -1 while. Peach bars and purple bars depict rG point estimate per trait for CanUD in EUR and AFR, respectively. Black bars represent standard error.

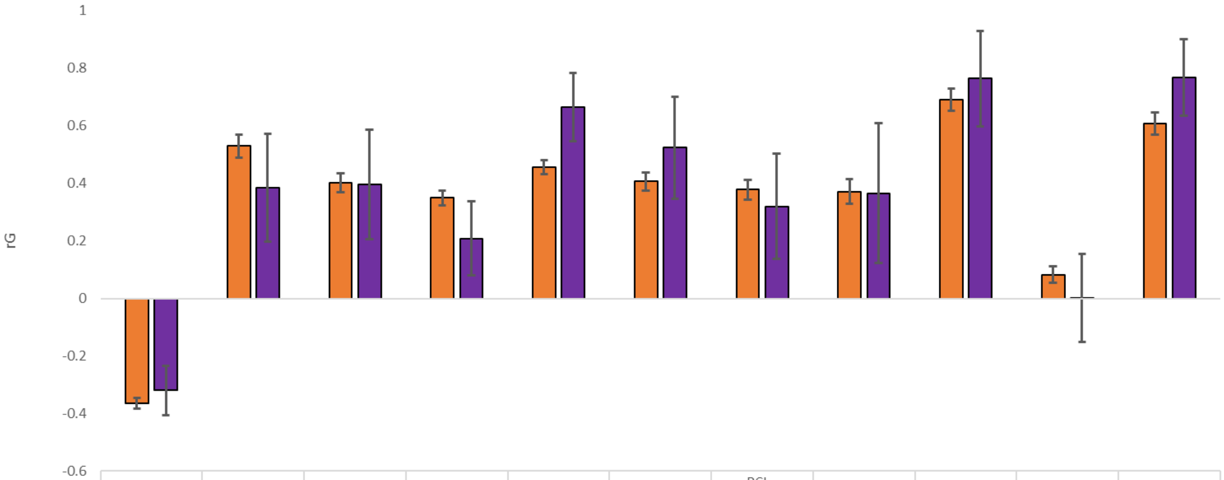

	EA	Unable to Work	PCL - Hyperarousal	Chronic Pain	MDD	PCL - Avoidance	PCL - Reexperiencing	Anxiety	Alcohol Use Disorder	Audit C	Smoking Initiation
CanUD EUR	-0.3632	0.5297	0.4019	0.3496	0.4565	0.4065	0.3779	0.3719	0.6908	0.0827	0.6082
CanUD AFR	-0.3193	0.3861	0.397	0.2082	0.6652	0.5236	0.3204	0.3654	0.764	0.0019	0.768

Supplementary Figure 4. ShinyGO dendrogram. Pathway enrichment.

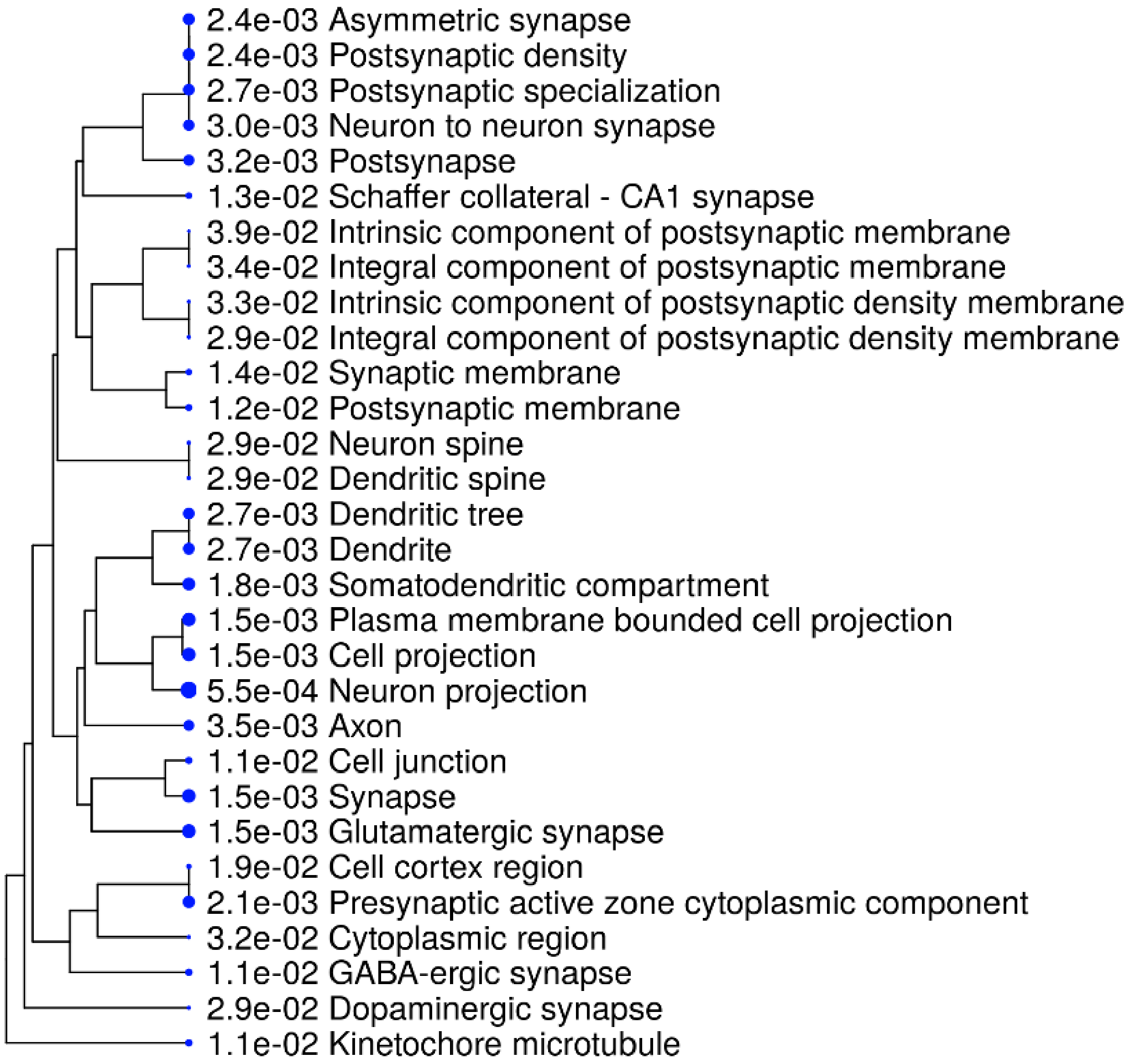

Supplementary Figure 5. MTAG plots for AUD. Tests were two sided. GWS threshold was used to determine significance (5x10^{-8}).

A

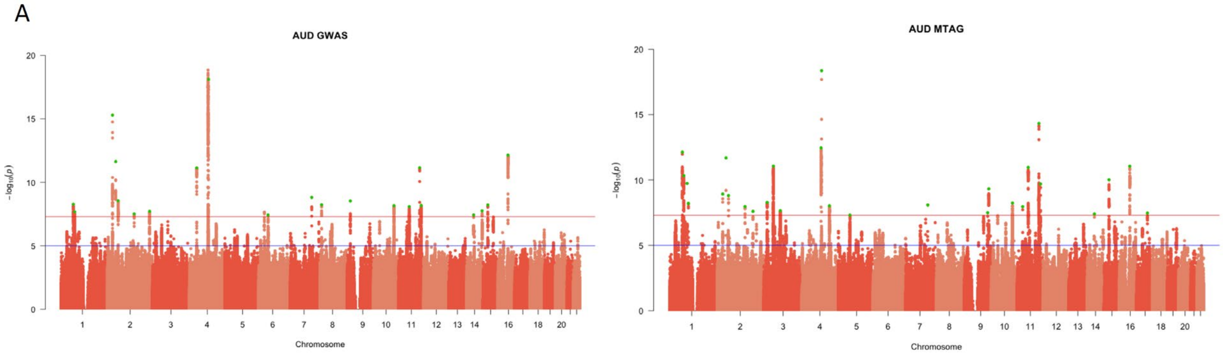

B

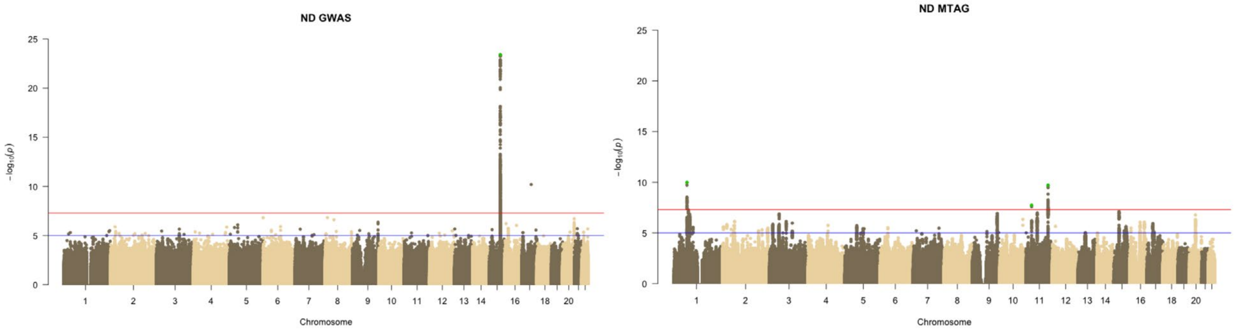

Supplementary Figure 6. GWAS Catalog Reported Gene Enrichment. Uncorrected p-value reported.

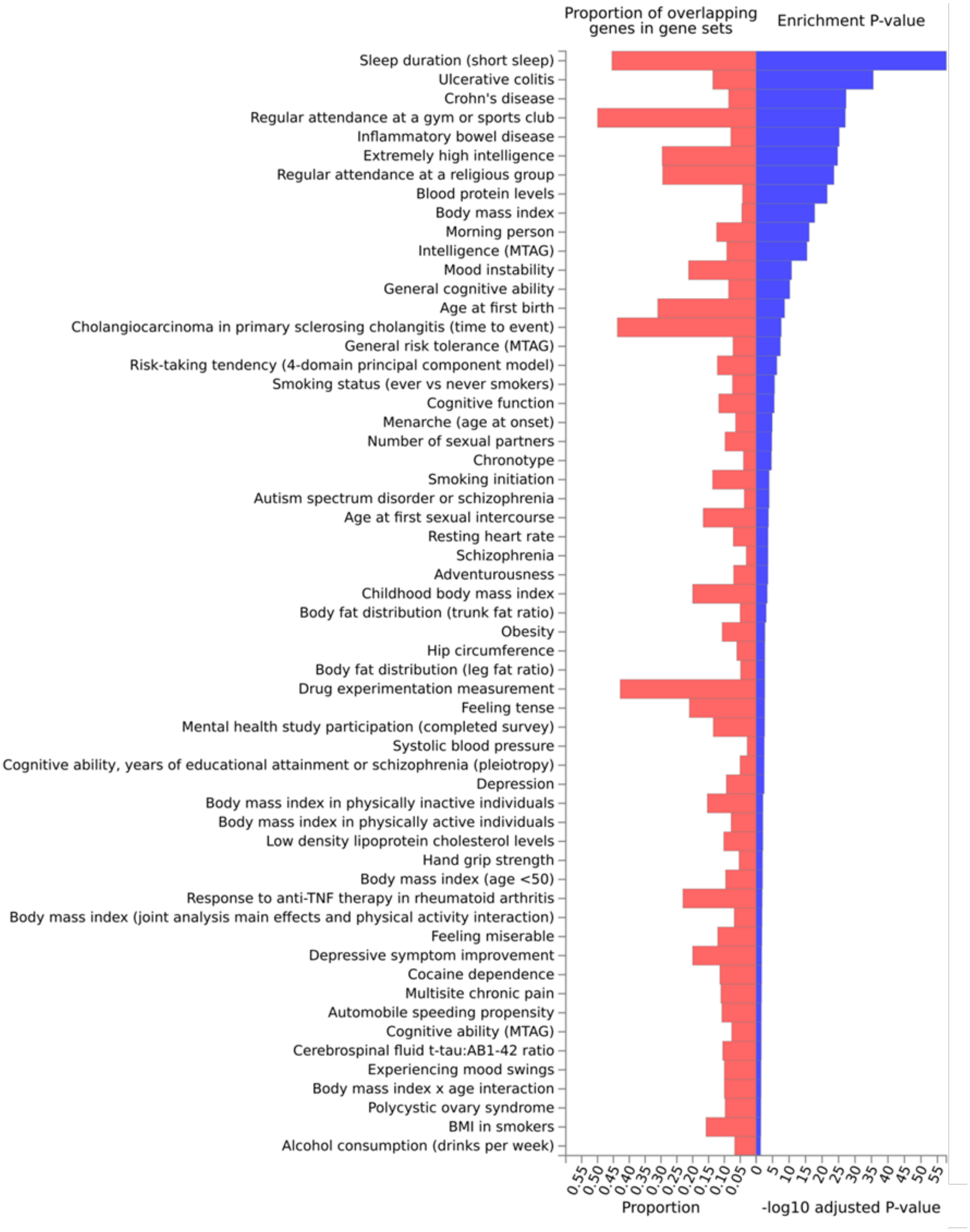

Supplementary Figure 7. Sex Stratified GWAS Within the MVP. Above, Female. Below, Male.

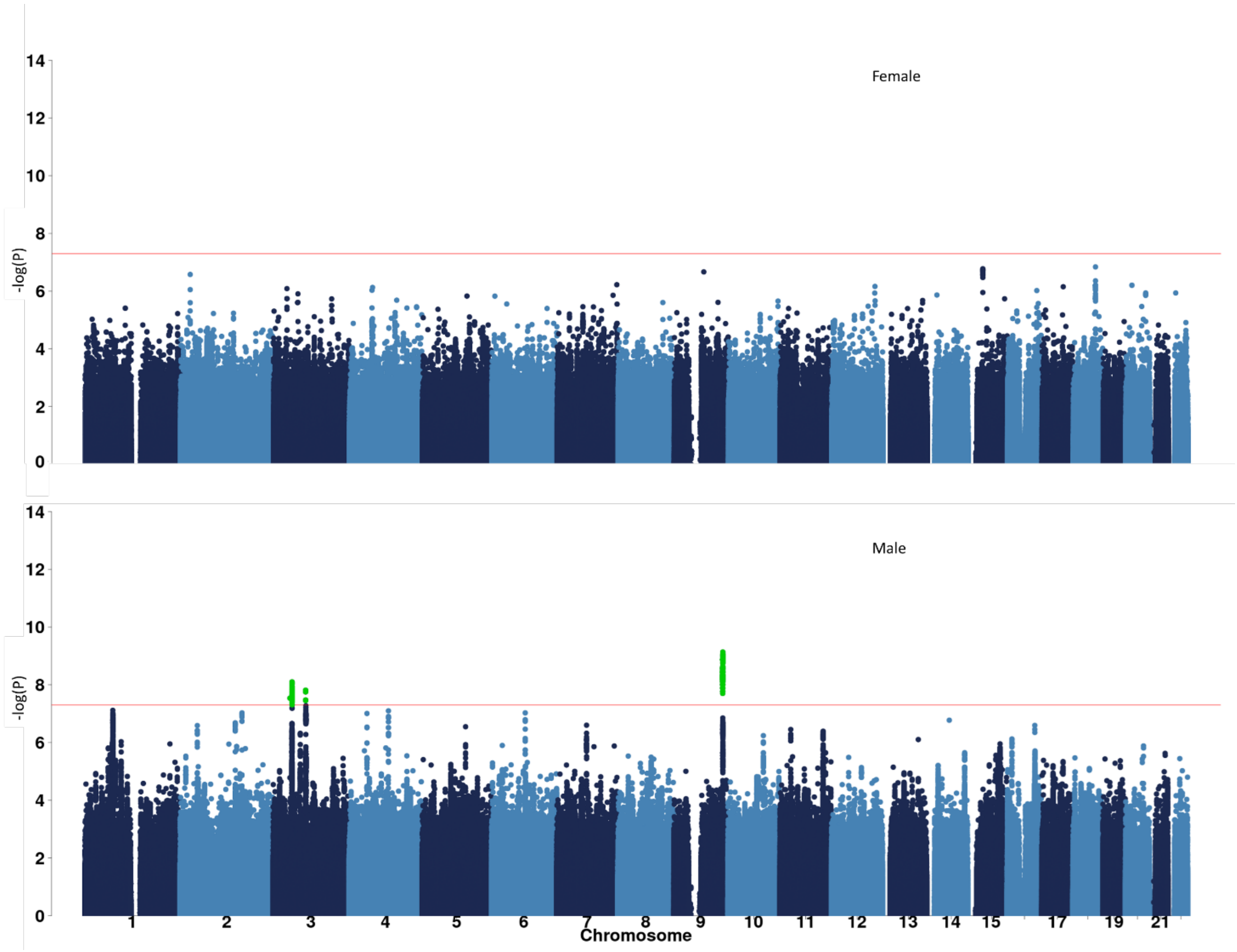

Supplement: Supplementary file 1 — Supplementary Tables 1–16 and Supplementary Figs. 1–7. [file 41588_2023_1563_MOESM1_ESM.pdf]
